# Natural and cross-inducible anti-SIV antibodies in Mauritian cynomolgus macaques

**DOI:** 10.1371/journal.pone.0186079

**Published:** 2017-10-05

**Authors:** Hongzhao Li, Mikaela Nykoluk, Lin Li, Lewis R. Liu, Robert W. Omange, Geoff Soule, Lukas T. Schroeder, Nikki Toledo, Mohammad Abul Kashem, Jorge F. Correia-Pinto, Binhua Liang, Nancy Schultz-Darken, Maria J. Alonso, James B. Whitney, Francis A. Plummer, Ma Luo

**Affiliations:** 1 Department of Medical Microbiology and Infectious Diseases, University of Manitoba, Winnipeg, Manitoba, Canada; 2 National Microbiology Laboratory, Public Health Agency of Canada, Winnipeg, Manitoba, Canada; 3 CIMUS Research Institute, University of Santiago de Compostela, Santiago de Compostela, La Coruña, Spain; 4 Department of Biochemistry and Medical Genetics, University of Manitoba, Winnipeg, Manitoba, Canada; 5 Wisconsin National Primate Research Center, Madison, Wisconsin, United States of America; 6 Center for Virology and Vaccine Research, Beth Israel Deaconess Medical Center, Harvard Medical School, Boston, Massachusetts, United States of America; 7 Ragon Institute of MGH, MIT, and Harvard, Cambridge, Massachusetts, United States of America; University of Pittsburgh Centre for Vaccine Research, UNITED STATES

## Abstract

Cynomolgus macaques are an increasingly important nonhuman primate model for HIV vaccine research. SIV-free animals without pre-existing anti-SIV immune responses are generally needed to evaluate the effect of vaccine-induced immune responses against the vaccine epitopes. Here, in order to select such animals for vaccine studies, we screened 108 naïve female Mauritian cynomolgus macaques for natural (baseline) antibodies to SIV antigens using a Bio-Plex multiplex system. The antigens included twelve 20mer peptides overlapping the twelve SIV protease cleavage sites (-10/+10), respectively (PCS peptides), and three non-PCS Gag or Env peptides. Natural antibodies to SIV antigens were detected in subsets of monkeys. The antibody reactivity to SIV was further confirmed by Western blot using purified recombinant SIV Gag and Env proteins. As expected, the immunization of monkeys with PCS antigens elicited anti-PCS antibodies. However, unexpectedly, antibodies to non-PCS peptides were also induced, as shown by both Bio-Plex and Western blot analyses, while the non-PCS peptides do not share sequence homology with PCS peptides. The presence of natural and vaccine cross-inducible SIV antibodies in Mauritian cynomolgus macaques should be considered in animal selection, experimental design and result interpretation, for their best use in HIV vaccine research.

## Introduction

Simian immunodeficiency virus (SIV) infection of nonhuman primates (NHPs) is currently the best animal model to test HIV vaccine strategies or study HIV pathogenesis [1–11]. Traditionally, rhesus macaques (*Macaca mulatta*) are the favorite choice among NHPs in HIV vaccine studies [1–5]. A wealth of knowledge has been accumulated for this species regarding SIV-host interaction, viral and cellular dynamics following SIV infection, genetics and physiology [6, 7]. However, the availability of rhesus macaques has been greatly reduced due to a ban of their export from India and most other south Asian countries [6, 12]. Cynomolgus macaques (*Macaca fascicularis*) have become by far the most internationally traded NHP for laboratory experiments [6]. In comparison to rhesus macaques, several characteristics make cynomolgus macaques a particularly useful animal model for HIV vaccine research, apart from their availability. SIV infection of cynomolgus macaques leads to a disease pattern that closely mimics that of human HIV infection, with lower peak and set-point viral loads and slower disease progression typical of human AIDS [6].

The largest laboratory supply of cynomolgus macaques is available from the island of Mauritius. The Mauritian cynomolgus macaques descended from a small group of founder animals and are characterized by high genetic homogeneity with much fewer MHC haplotypes and alleles [6, 7, 13–15]. This helps reduce outbred variability between animals and thus reduces the number of animals needed to achieve statistical power, making them practical for HIV vaccine studies [7].

Commonly, vaccine studies are carried out in specific pathogen-free animals to rule out the impact of on-going infection or pre-existing immune responses in order to soley evaluate the vaccine efficacy absent of confounding variables. This may require screening larger numbers of animals than those used in the vaccine experiments. Published reports from HIV vaccine studies using NHPs generally failed to provide details of the cohort screening. However, such information is valuable and can serve to guide future vaccine projects. A practical concern in vaccine studies is that a large number of animals are required to achieve statistical power. The information of expected frequency of animals with pre-existing infection or immune responses is important for estimating the starting number of animals to be screened. Here, we report the levels and frequencies of natural antibodies to SIV antigens among a population of 108 Mauritian cynomolgus macaques. These SIV antigens include peptides surrounding the twelve protease cleavage sites [16] (PCS peptides) and three non-PCS Gag or Env peptides of SIVmac239 [17–19]. In addition, we observed that vaccination of Mauritian cynomolgus macaques with PCS antigens not only elicited antibodies to the PCS peptides, but also cross-induced antibodies to non-PCS peptides, while the non-PCS peptides share no sequence homology with the PCS peptides. This suggests that a vaccine could elicit immune responses targeting SIV antigens other than those directly from the vaccine. These novel antibody responses need to be taken into consideration in HIV vaccine projects using Mauritian cynomolgus macaques.

## Materials and methods

### Experimental animals and ethics statement

108 female Mauritian cynomolgus macaques (*Macaca facicularis*) from Bioculture (Mauritius) Ltd were involved in this study, including groups of 94 colony-bred animals and 14 capture-bred animals. From the former group 94 animals only their plasma samples were purchased for *in vitro* antibody analysis, without any physical involvement of these animals in this study. Only the latter group 14 animals were physically involved in the animal work of this study and were used for immunization and viral challenge experiments. The immunization and viral challenge experiments are detailed below as part of *Materials and Methods*. The animal work was conducted in accordance with Canadian Council on Animal Care guidelines and the Animal Use Document was approved by the Canadian Sciences Centre for Human and Animal Health Animal Care Committee (protocol number: H-12-014R2). The humane care of animals was performed as previously described [20]: "Animals were double housed in standard non-human primate cages, received standard primate feed as well as fresh fruit and enrichment daily, and had continual access to water. Temperature (19–24°C), humidity (45–60%) and light (approximately 323 lux) were monitored and maintained within recommended limits, the light/dark cycle was maintained at 12 hour split. Environmental enrichment was provided. Animals were observed twice daily by the PI, a co-investigator, or the veterinary staff for signs of clinical illness”. The cages meet CCAC guidelines for primate housing. The dimensions of the one-over-one primate cages used measure at 38 inches wide, 49 inches deep and 101 inches high. Anesthesia was administered to alleviate suffering by injection with Ketamine Hydrochloride at a dosage of 10 mg/kg (i.m., using 23–25 gauge, 3/8-1 inch needles) prior to phlebotomy or intravaginal viral challenge. Animals were sacrificed at the end of the study or in situations of multiple systemic consequences from simian AIDS, such as wasting (weight loss), diarrhea, generalized lymphadenopathy, pneumonia, encephalitis, vascular thrombosis and secondary infections coupled with complete anorexia for more than two days resulting in acute weight loss of more than 20%. Following induction of deep anesthesia with Ketamine Hydrochloride at a dosage of 25–50 mg/kg (using 23–25 gauge, 3/8-1 inch needles), euthanasia was performed by terminal bleeding (femoral and/or intracardiac exsanguination). Sedation was maintained by inhalation of Isoflurane 2.5–3.5% by mask in 100% oxygen.

### The SIV antigens

During HIV or SIV replication, each of the 12 protease cleavage reactions is essential for the production of a functional viral particle [16]. A novel vaccine strategy targeting the protease cleavage sites (PCS) has been suggested by our studies [16, 21] and is being evaluated using Mauritian cynomolgus macaque SIV infection model. As part of the ongoing work, we used PCS peptide antigens (along with non-PCS peptides) to screen for potentially pre-existing natural antibody responses in Mauritian cynomolgus macaques, while no natural immune response screen study has been reported in these animals. Specifically, the SIV antigens used in this study were twelve 20mer peptides overlapping the twelve PCS (-10/+10) and three non-PCS Gag or Env peptides, derived from SIVmac239 [17–19] (Table 1). The sequences of all these peptides were confirmed to be specific for SIV by NCBI protein BLAST and are conserved among multiple SIV strains (Data not shown). No sequence homology was shared between PCS versus non-PCS peptides (Figure A in S1 File).

**Table 1:** SIV antigen peptides used in antibody screening.

PCS or non-PCS	SIV protease cleavage location	Sequence
PCS1	p15(MA)/p27(CA)	APSSGRGGNY/PVQQIGGNYV
PCS2	p27(CA)/p2	GGPGQKARLM/AEALKEALAP
PCS3	p2/p8(NC)	LAPVPIPFAA/AQQRGPRKPI
PCS4	p8(NC)/p1	MAKCPDRQAG/FLGLGPWGKK
PCS5	p1/p6gag	GPWGKKPRNF/PMAQVHQGLM
PCS6	Ncgag-pol/TFP	YGQMPRQTGG/FFRPWSMGKE
PCS7	TFP/p6gag-pol	WSMGKEAPQF/PHGSSASGAD
PCS8	p6gag-pol/p10(PR)	LQGGDRGFAA/PQFSLWRRPV
PCS9	p10(PR)/p66(RT/RNase)	LTALGMSLNF/PIAKVEPVKV
PCS10	p51(RT)/p15(RNase)	KDPIEGEETY/YTDGSCNKQS
PCS11	p66(RT/RNase)/p31(IN)	LVSQGIRQVL/FLEKIEPAQE
PCS12	Nef	NQGQYMNTPW/RNPAEEREKL
SIVgag	No cleavage	VGDHQAAMQIIRDIINEEAADWDL
SIVenv1	No cleavage	NVTESFDAWNNTVTEQAIEDVWQLFETSIRPCVKLSP
SIVenv2	No cleavage	RVTAIEKYLKDQAQLNAWGCAFRQVCHTTVPWPNA

### Bio-Plex multiplexed antibody assay

Plasma IgG antibodies to SIV antigens were quantified by largely following the previously published protocols [22, 23] with slight modifications. Briefly, 20 μg of antigen peptide (synthesized by Genscript, Piscataway, NJ) was coupled to 1.25 x 10^6^ Bio-Plex Pro^™^ Magnetic COOH Beads (Bio-Rad) using a Bio-Plex Amine Coupling Kit (Bio-Rad). 50 μl plasma (1:80 diluted) was incubated with 2,500 beads/antigen type. SIV-specific IgG was detected with phycoerythrin-labelled mouse anti-monkey IgG (Southern Biotech, Birmingham, AL) at 5 μg/ml. Bead fluorescence intensities were acquired on the Bio-Plex 200 system (Bio-Rad) and converted to concentrations based on estimation using a PCS2 monoclonal antibody (National Microbiology Laboratory, Canada) as standard.

### Viral challenge and antiretroviral drug treatment (ARV)

Five macaques (previously immunized with a vesicular stomatitis virus vaccine vector, rVSVwt) were intravaginally challenged with 1000 TCID_50_ SIVmac251 (Desrosiers 2010-Day 8 viral stock, provided by Drs. Jon Warren, and Nancy Miller, Vaccine Research Program, NIH). The challenge was repeatedly carried out (ranging two to four times) in each animal until positive plasma viral load (VL) was detected. The VL was then monitored weekly throughout the experiment. Daily antiretroviral drug treatment with a combination of FTC (50mg/kg body weight), PMPA (20mg/kg) and raltegravir (10mg/kg) was initiated 25 weeks after SIVmac251 infection. Plasma IgG antibodies to SIV peptides were quantified by Bio-Plex multiplexed antibody assay for time points of weeks 0 and 5 post ARV initiation.

### Viral load assay

This was conducted following a previously published protocol [20].

### Indirect enzyme-linked immunosorbent assay (ELISA) quantification of antibodies to vesicular stomatitis virus (VSV) and Zaire Ebola virus (ZEBOV)

96-well ELISA plates were coated with 100 μl of 1 μg/ml purified VSV or ZEBOV [24] (National Microbiology Laboratory, Canada) as capture antigens at 4°C overnight. Plate washing along the ELISA procedure was performed with PBS containing 0.1% Tween20 and blocking was with 5% skim milk in PBS containing 0.1% Tween20. Monkey plasmas diluted at 1:100 and a following HRP-conjugated goat anti-human IgG secondary antibody (Kirkegaard & Perry Laboratories, catalog number 074–1006) diluted at 1:2000 were used, respectively, both at 37°C for 1 hour. After a final incubation with a TMB substrate solution (ThermoFisher Scientific, catalog number 34028) at 37°C for 30 minutes, optical density (OD) values were read at 650 nm.

### MHC genotyping

The Cynomolgus macaque MHC haplotype typing was conducted by Wisconsin Nonhuman Primate Research Centre Genetics Services [25, 26].

### Construction of recombinant VSV vectors encoding the nucleotide sequences of the 12 protease cleavage sites (rVSVpcs vectors)

The sequence of Simian immunodeficiency virus strain SIVmac239 was retrieved from the Los Alamos National Laboratory HIV database (http://www.hiv.lanl.gov). The nucleotide sequences encoding 20 amino acids (10 amino acids flanking each side of the cleavage site) overlapping each of the 12 PCS [MA(p15)/CA(p27), CA(p27)/p2, p2/NC(p8), NC(p8)/p1, p1/p6gag, NCgag-pol/TFP, TFP/NCgag-pol, p6gag-pol/Protease(p10), Protease(p10)/ RT(p66), RT(p51)/RNase(p15), RNase(p15)/integrase (p31) and Nef] (Table 1) were synthesized and cloned in a Blue Heron pUC(-)MCS plasmid (BlueHeron Biotechnology, Bothell, WA, USA). Each PCS sequence was flanked by an upstream MluI restriction site (AAACGCGT), Kozak sequence (GCCACC), start codon, and downstream stop codon and AvrII restriction site (CCTAGGTT). The PCS coding fragment was then sub-cloned into a modified Vesicular Stomatitis Virus (VSV) vector, a gift to Gary Kobinger from John Rose, Yale University School of Medicine. The modified VSV plasmid (pATX VSV-G) expresses the positive-strand RNA complement of the VSV genome and can tolerate the addition of four foreign genes at four multiple cloning sites (MCS #1–4). MCS#3 was identified to promote the highest expression levels of luciferase and EGFP reporter genes by luciferase assays and FACS analysis (Wong 2011, personal communications). The pATX VSV-G vector contained in order: bacteriophage T7 promoter (T7P), the VSV leader, nucleoprotein (N), phosphoprotein (P), matrixprotein (M), glycoprotein (G) (in MCS#4), polymerase (L), ampicillin resistance gene (AmpR), hepatitis delta virus ribozyme and the T7 terminator sequence. Sub-cloning of PCS coding sequences into MCS#3 of pATX VSV-G was performed using the following procedure. pATX-VSV-G vector and Blue Heron pUC(-)MCS plasmids containing each of the twelve PCS were double digested with AvrII and MluI (New England BioLabs, ON, Canada). 20 ul of each digested product was electrophoresed in a 1% low-melt agarose gel (Invitrogen, CA, USA) and stained with SYBR^®^ Gold (Invitrogen, CA, USA) for 15 minutes. The linearized pATX VSV-G vector band was excised and purified using the QIAquick Gel Extraction kit (Qiagen, USA). The PCS DNA band from each digested Blue Heron pUC(-)MCS plasmid was excised and purified using the QIAEX II Agarose Gel Extraction kit (Qiagen, USA). Each of the 12 PCS fragments was cloned into the digested pATX VSV-G vector by ligation with T4 DNA ligase (Invitrogen, CA, USA). Ligated plasmids were transformed into One Shot^®^ Top10 cells (Invitrogen, CA, USA) and purified following the Endofree Plasmid Maxi purification kit (Qiagen, USA). All recombinant VSV plasmids were verified by sequencing (DNA Core Facility, National Microbiology Laboratory, Canada) using BigDye Terminator Cycle Sequencing Ready Reaction kits (Applied Biosystems, Foster City, CA) and Prism 3130xl Genetic Analyzer (Applied Biosystems, Foster City, CA).

### rVSVpcs virus generation

HEK293T and VeroE6 cells were grown in Dulbecco’s modified Eagle’s medium (DMEM) containing 10% fetal bovine serum (FBS), penicillin (100 U/ml), streptomycin (100 μg/ml), L glutamine (2mM) (Invitrogen, CA, USA). Cells were transfected with rVSVpcs plasmid (2 μg) and support plasmids (2 μg of T7, 0.5 μg of N, 0.3 μg of L and 1.3 μg of P) with Lipofectamine 2000 (Invitrogen, CA, USA) according to manufacturer’s protocols. Cells were incubated at 37°C/5% CO_2_ until the transfected cells displayed cytopathetic effect compared to negative control cells. Rescued rVSVs were then passaged on VeroE6 cells to obtain a virus stock. Virus stock was purified and concentrated by ultracentrifugation through a 20% sucrose cushion in a Beckman XPN-80 ultracentrifuge at 27,000 RPM, 4°C for 2 hours. The final pellet was resuspended in DMEM and stored at -80°C. Purified rVSVpcs stock was plaque titrated with VeroE6 cells.

### Packaging of PCS peptides into nanoparticles (NANOpcs)

Twelve 20mer peptides overlapping the PCS of SIVmac239 were synthesized (GenScript) for nanopackaging. The peptides were associated to a nanoparticle system formed by chitosan (CS) and dextran sulfate (DS). Due to their opposite charges, it is possible to form nanoparticles by a simple ionic interaction process under mild conditions. Briefly, the CS:DS nanoparticles were formed spontaneously upon addition of 0.825 ml of an aqueous DS solution (1.875 mg/ml) to the same volume of an aqueous CS solution (0.625 mg/ml) under magnetic stirring. For peptide encapsulation, the peptide was incorporated in the anionic (DS solution) or in the cationic phase (CS solution) according to its isoelectric point (pI). Peptides with pI lower than 7, were dissolved in the anionic phase while the rest of the peptides were dissolved in the cationic phase. The peptide theoretical loading in nanoparticles was 9.6%. Each one of the peptides was encapsulated separately, and then, a pool of loaded nanoparticles, containing 50 μg of each peptide, was prepared in individual vials for a single administration. For an improved conservation, the nanoparticles suspension in each vial, were incorporated in a cryoprotectant solution (trehalose 5%, w/v) and submitted to a freeze-thaw cycle. The vials were frozen at -80°C overnight and then submitted to two sequential drying steps in a lyophilizer. The primary drying was carried out at -35°C (40 h) under high vacuum, and then the second drying step (8 h), in which the temperature gradually raised until +20°C, forming an uniform dry cake. To evaluate the effect of this operation over the formulation characteristics, loaded nanoparticles with each peptide, were freeze-dried with the same protocol and afterwards reconstituted with ultrapure water in order to analyze their size and polydispersity index (PdI). The nanoparticles diameter and PdI were evaluated by diffraction laser spectroscopy and its surface charge by electrophoretic mobility using a Zetasizer Nano ZS90 (Malvern Instruments, UK). The morphology of the particles was evaluated by transmission electronic microscopy (TEM). Ultrapure CS hydrochloride salt, having a molecular weight of around 125 kDa and an acetylation degree of 14% (Protasan UP Cl 113), was purchased from Novamatrix (Norway). The DS with a molecular weight of about 14,500 Da was obtained from Sigma–Aldrich (Madrid, Spain).

### Immunization: PCS vaccination

Fourteen monkeys were immunized (i.m., right hind leg). Each of 8 monkeys in the vaccine group received a total of 1.2×10^7^ plaque forming units (pfu) of pooled rVSVpcs viral particles (1×10^6^ pfu for each of the 12 rVSVpcs). Each of the 6 monkeys in the control group received 1.2×10^7^ pfu of rVSV wild type (rVSVwt). The monkeys were boosted at week 5, with PCS peptides packaged in nanoparticles (NANOpcs) for vaccine group and water (NANO vehicle) for control group, and boosted again at week 9, with rVSVpcs/NANOpcs for vaccine group and rVSVwt/water for control group.

### Enrichment of SIV peptide-specific antibodies

SIV peptides (synthesized by Genscript, Piscataway, NJ) were coupled to CarboxyLink coupling gel (ThermoFisher Scientific, Rockford, IL; Catalog 20266) and used to enrich SIV peptide-specific antibodies from crude monkey plasma. Total plasma antibodies were purified using Pierce protein A/G agarose beads (ThermoFisher Scientific, Catalog 20422).

### Western blot

SDS-PAGE was conducted following the NuPAGE Bis-Tris mini gel electrophoresis protocol (Thermo Fisher Scientific, Waltham, MA). Purified recombinant SIV proteins (NIH AIDS Reagent Program), SIVmac251 Gag (Catalog 1845) and SIVmac239 Env (Catalog 2322) were diluted in 1× NuPAGE LDS sample buffer (Thermo Fisher Scientific, Catalog NP0008) containing 1× NuPAGE reducing agent (Thermo Fisher Scientific, NP0009) and heated at 70°C for 10 minutes. 10 μl molecular weight marker, Precision Plus protein Dual Color Standards (Bio-Rad, 161–0374), Magic Mark XP Western Standard (Thermo Fisher Scientific, LC5602) or Spectra^™^ Multicolor Broad Range Protein Ladder (Thermo Fisher Scientific, 26634), or 1 μg SIV protein in 15 μl sample buffer was loaded onto a NuPAGE 4–12% Bis-Tris 1.0mm×10well gel (Thermo Fisher Scientific, NP0321BOX) assembled in a mini gel tank (Thermo Fisher Scientific, A25977). The buffer chambers were filled with 1×NuPAGE MES SDS running buffer (Thermo Fisher Scientific, NP0002). NuPAGE^™^ Antioxidant (Thermo Fisher Scientific, NP0005) was added to the running buffer of the upper (cathode) chamber at 400× dilution. The electrophoresis was run at 200V for 35 minutes. Blotting was performed using iBlot gel transfer device (Thermo Fisher Scientific, IB1001) and iBlot gel transfer nitrocellulose mini stacks (Thermo Fisher Scientific, IB301002), according to the supplier’s protocol. Blotting membranes were rinsed with wash buffer (PBS containing 0.1% Tween 20) and then blocked with 5% skim milk in wash buffer at room temperature with shaking at 100 rpm. 3× wash of 5 minutes at room temperature with shaking was performed after blocking and between/after antibody incubation steps. Two types of primary antibodies were used. To confirm if natural SIV peptide-specific antibodies recognize authentic SIV proteins, antibodies enriched from monkey plasma (as described above) were diluted at 1 μg/ml in antibody buffer (wash buffer containing 0.5% skim milk). Control antibodies were protein A/G-purified total Ig from SIV antibody-negative monkey plasma. To confirm if PCS vaccination-induced monkey antibodies recognize authentic SIV proteins, monkey crude plasmas from time points of baseline or 1 week after PCS vaccination were diluted at 1:125 in antibody buffer. In both cases, the diluted primary antibodies were incubated with membranes at 4°C with shaking overnight. The secondary antibody, goat anti monkey IgG-HRP (Santa Cruz Biotechnology, sc-2458) was diluted at 1:2000 in antibody buffer and incubated with membrane for 1 hour at room temperature with shaking. Chemiluminescent detection was based on Pierce ECL Western blotting substrate (Thermo Fisher Scientific, 32209) and carried out on a ChemiDoc XRS instrument using Quantity One 4.6.9 software (Bio-Rad). The application setting was chemi Hi sensitivity, no light 2×gain, 2×2 Bin and manual exposure for 5 minutes. To control sample loading, membranes were stripped in Restore^™^ PLUS Western blot stripping buffer (Thermo Fisher Scientific, 46428) for 15 minutes at room temperature with shaking and re-probed with standard SIV antibodies. The standard SIV antibodies were a mixture of multiple clones of NIH AIDS Reagent Program mouse monoclonal antibodies specific for SIV Gag or Env proteins. For Gag, the antibodies include catalog numbers of 2320 and 2321, which were each 1:4,000 diluted. For Env, the antibodies include catalog numbers of 4669, 2695, 2319, 2696, 12224, 2317, 4670, 1403, 12223 and 1812, which were each 1: 20,000 diluted. The secondary antibody in combination was goat anti-mouse IgG-HRP (Santa Cruz Biotechnology, sc-2005), used at 1:5,000 dilution. To quantify vaccine-induced changes in the levels of monkey plasma IgG antibodies to SIV Gag or Env proteins, the SIV antibody levels were defined by normalizing the band intensities in the initial monkey antibody blots to their counterparts in the re-probed, mouse antibody blots.

### Statistical analysis

Statistical analysis was performed using GraphPad Prism statistical software. Continuous variables were compared with Student's *t* test; categorical variables were compared with Fisher's exact test.

## Results

### Natural antibodies to novel SIV antigens are detected in naïve Mauritian cynomolgus macaque populations

**Fig 1:**
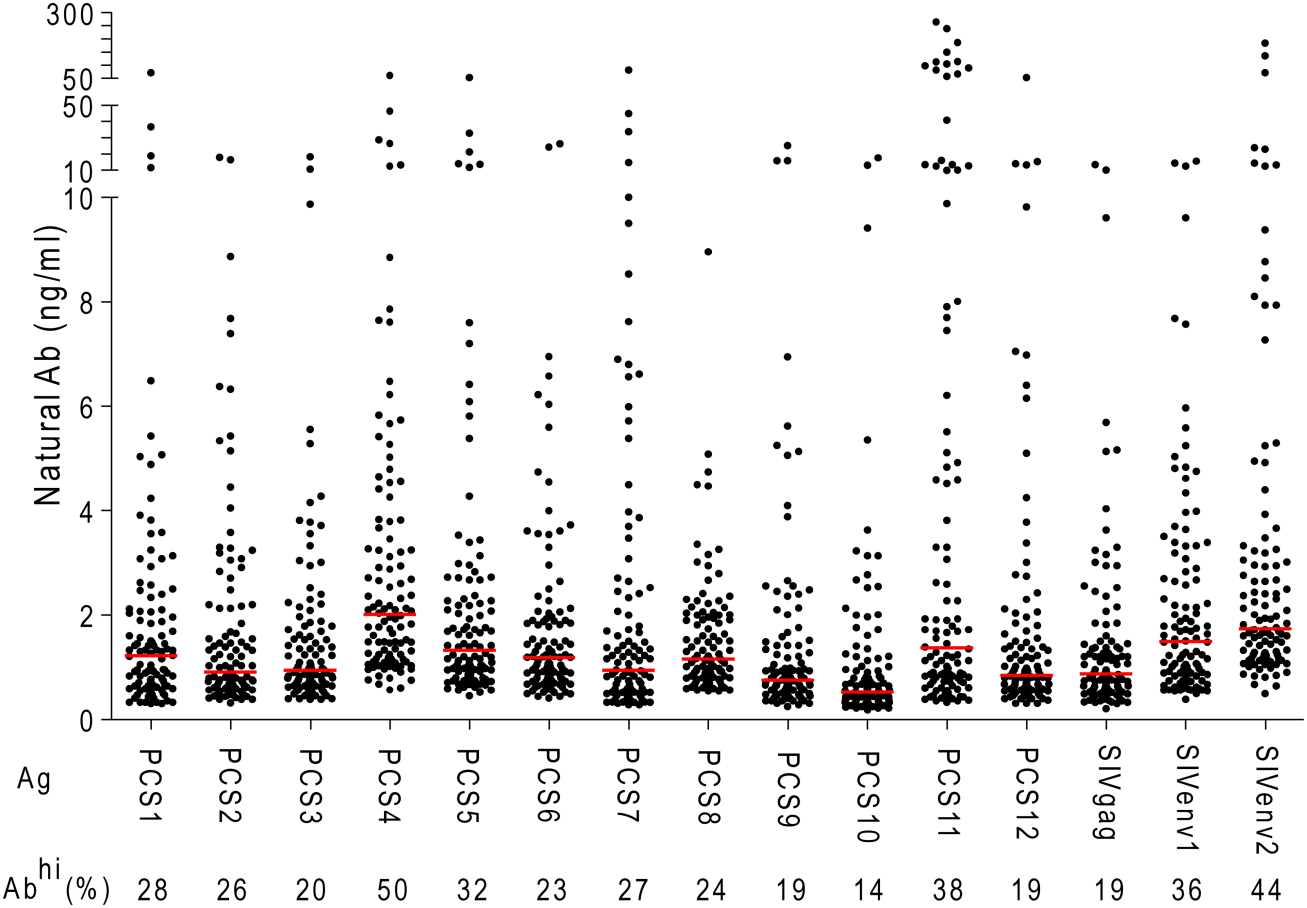
Natural antibodies to SIV peptide antigens in Mauritian cynomolgus macaques. 108 naïve healthy female Mauritian cynomolgus macaques maintained in SIV-free facilities were analyzed by Bio-Plex for plasma IgG antibodies to SIV peptide antigens (Ag). Each black dot indicates one monkey. Red lines represent medians of antibody levels. Ab^hi^: antibody level ≥ 2 ng/ml. Frequency of Ab^hi^ monkeys was listed for each Ag type.

**Fig 2:**
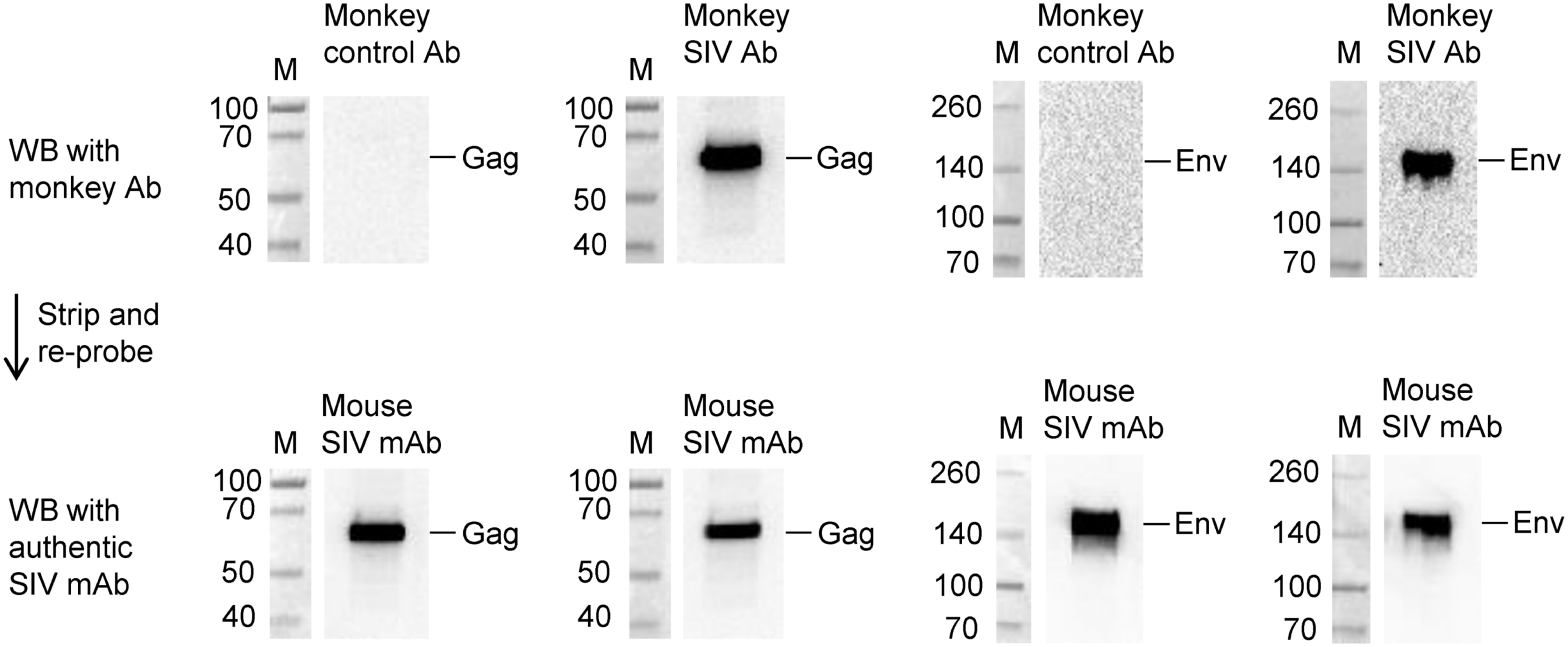
Natural antibodies to SIV peptides recognize authentic SIV proteins. The reactivity of SIV peptide-specific antibodies to SIV proteins was evaluated by Western blot (WB) analysis. Antigens used on these blots were purified recombinant SIV Gag or Env proteins (NIH AIDS Reagent Program). Plasma antibodies to SIV peptides as in Fig 1 were enriched using SIV peptide-coupled affinity columns. The enriched antibodies (Monkey SIV Ab) were then tested for reactivity against Gag or Env proteins. Control antibodies (Monkey control Ab) were protein A/G column-enriched total antibodies from monkey plasma negative (low) for SIV peptide-specific antibodies. M, molecular weight markers (KDa). The monkey antibody blots (upper row) were stripped and re-probed (lower row) with authentic SIV antibodies, mouse anti-Gag and Env monoclonal antibodies (Mouse SIV mAb). Blots shown represent three experiments with the same results.

**Fig 3:**
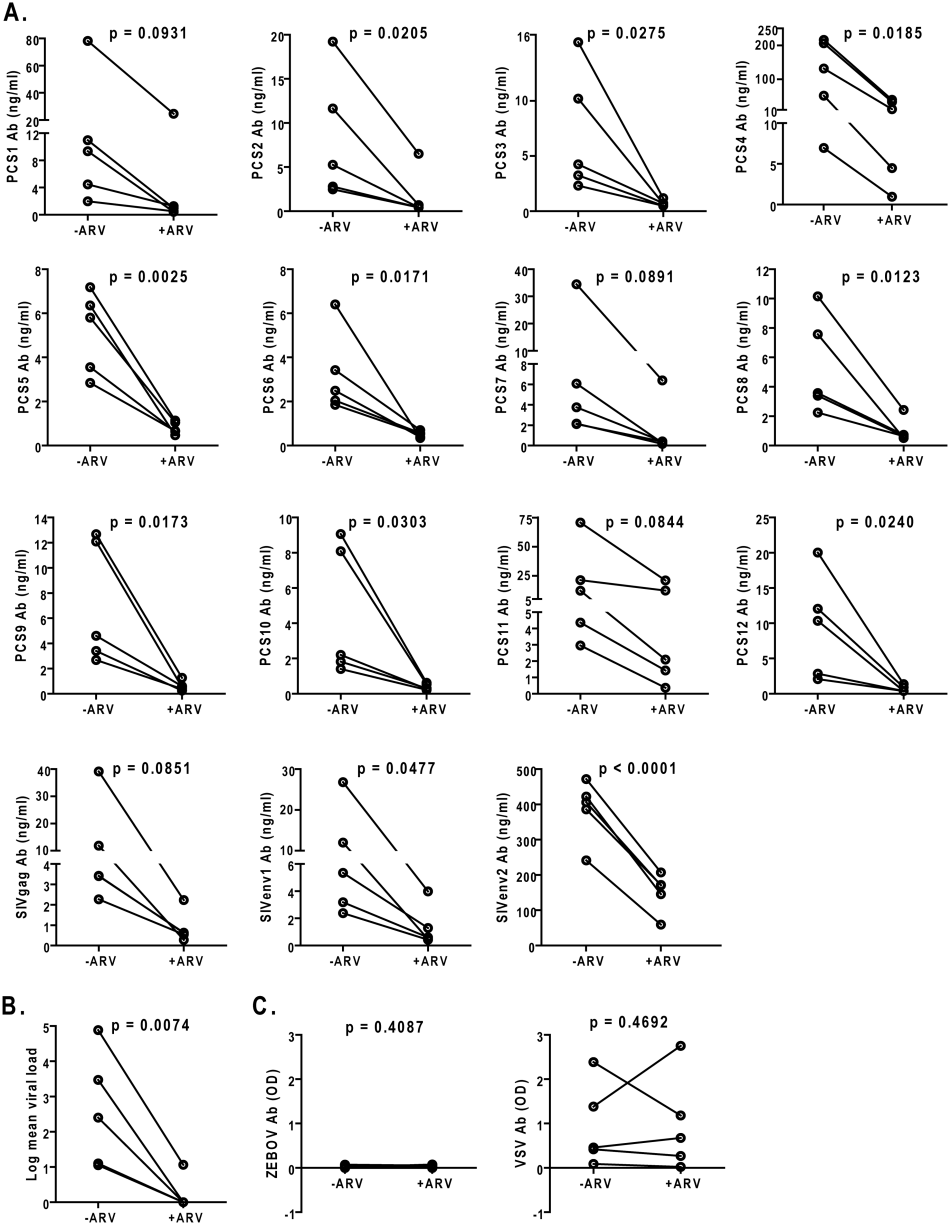
Confirmation of antibodies to the SIV peptide antigens as anti-SIV antibodies in the context of SIV infection and anti-retroviral treatment. (A) 5 monkeys were used in SIV infection and anti-retroviral drug (ARV) treatment experiments. They had previously been immunized with a vesicular stomatitis virus (VSV) vector, and had not been infected by other viruses including SIV and Zaire Ebola virus (ZEBOV). Here, they were experimentally infected with SIVmac251, maintained for 25 weeks and then treated daily with anti-retroviral drugs (ARV). Plasma IgG antibodies to SIV peptide antigens were quantified by Bio-Plex for time points before and after ARV treatment, at week 0 (-ARV) and week 5 (+ARV) post ARV initiation, respectively. Each line represents one monkey, connecting data points before and after ARV. Student’s paired *t* tests were conducted to assess the reduction of SIV antibodies by ARV. (B) Plasma SIV viral loads of the five monkeys in A before and after ARV. Viral load reduction by ARV was confirmed by Student’s paired *t* test. (C) Levels of plasma IgG to Zaire Ebola virus (ZEBOV) and vesicular stomatitis virus (VSV) of the five monkeys in A were quantified by ELISA. OD, optical density. No significant difference was found in either case between before and after ARV by Student’s paired *t* test.

To prepare for a preclinical evaluation of a novel HIV vaccine strategy targeting the 12 viral protease cleavage sites (PCS) using an intravaginal challenge model, we screened 108 randomly selected, naïve female Mauritian cynomolgus macaques for potential natural antibodies against SIV antigens, including 12 PCS peptides (our vaccine of interest) and 3 non-PCS Gag and Env peptides (See Materials and methods for details). The macaques, 14 capture-bred and 94 colony-bred, had not been used in any previous SIV challenge or vaccination study before the antibody screening. Using a Bio-Plex multiplexed antibody assay, we found that subsets of animals had higher than background levels of antibodies (Fig 1 and Table A in S1 File). Since these monkeys had not been previously infected with SIV or exposed to SIV antigens that would induce SIV antibodies, we carefully validated the results obtained with the Bio-Plex method. We used two other methods to verify whether these are anti-SIV antibodies. We first tested whether these antibodies recognize authentic SIV proteins by Western blot analysis using affinity-purified SIV peptide-specific antibodies from monkey plasma samples. The results showed that these antibodies indeed recognized purified recombinant SIV Gag and Env proteins (Fig 2). Next, we characterized antibody responses to these SIV peptide antigens in the scenario of SIV infection (Fig 3). Five monkeys were experimentally infected with SIVmac251, followed by treatment with anti-retroviral drugs (ARV) FTC, PMPA and raltegravir [27, 28]. We found that the levels of antibodies to PCS and non-PCS peptides were high before ARV treatment, but were significantly decreased after ARV (Fig 3A) and the decrease of antibodies to the SIV peptide antigens corresponded to the decrease of SIV viral load (Fig 3B). To exclude the possibility that the reduction of anti-SIV antibody responses might be due to a general impairment by ARV in the host antibody production function, we evaluated anti-vesicular stomatitis virus (VSV) antibodies in these animals as they had previously been immunized with a VSV vector (rVSVwt). We found that their anti-VSV antibody responses were not inhibited by ARV (Fig 3C). Thus, a potential non-specific effect of ARV was ruled out. Together, these results confirmed that the antibodies to PCS and non-PCS peptide antigens are indeed anti-SIV antibodies.

### Possible factors influencing the level of natural antibodies to SIV antigens

**Fig 4:**
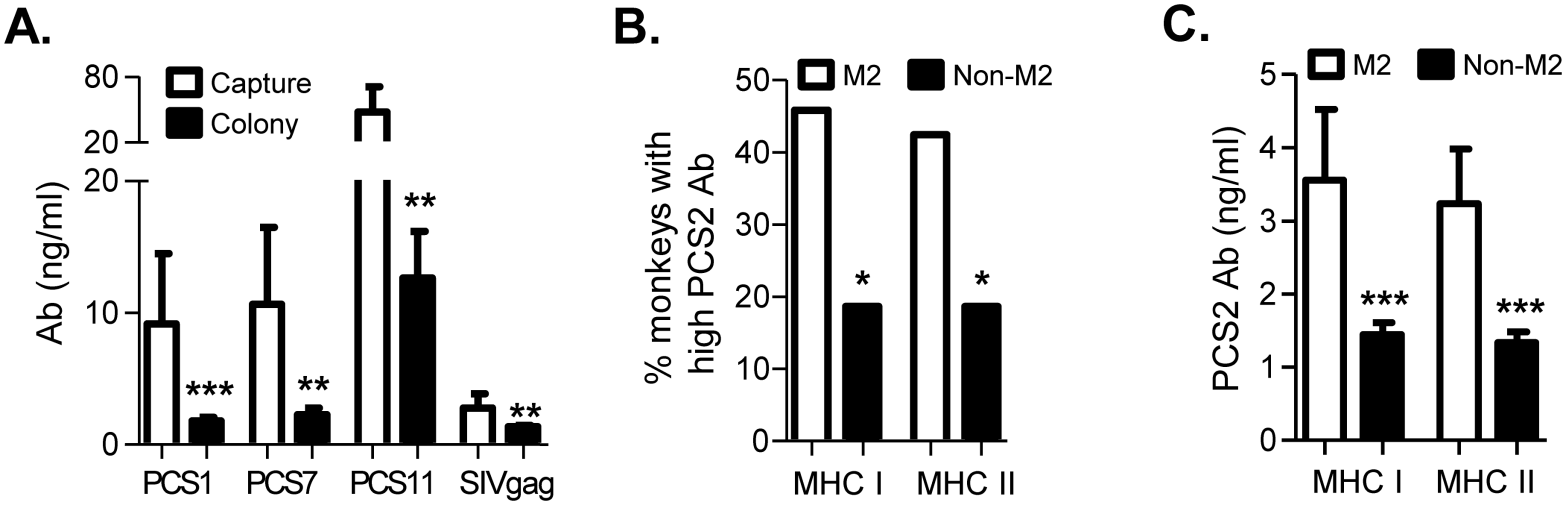
Environment and host factors may have an impact on natural antibody responses to SIV antigens. (A) Natural antibody responses to SIV antigens were compared between capture bred (14 animals) and colony bred (94 animals) monkeys. Data are mean ± SEM of plasma IgG concentrations. Significant difference between the two sub-groups was determined for antibodies to PCS1, PCS7, PCS11 and SIVgag by Student’s *t* test: *** p < 0.001 and ** p < 0.01. No difference was found for antibodies to the other antigen types (data not shown). (B) MHC I M2 and MHC II M2 haplotypes correlate with higher frequencies of monkeys with stronger natural antibody responses to PCS2. Bars represent frequencies of monkeys with ≥ 2 ng/ml plasma IgG to PCS2 in those with or without M2 haplotype of MHC I or MHC II. Correlation analysis was performed using Fisher’s exact test: * p < 0.05. (C) MHC I M2 and MHC II M2 haplotypes correlate with higher levels of natural antibodies to PCS2. Bars represent mean ± SEM of concentrations of plasma IgG to PCS2. Significant difference between MHC I M2 and MHC I non-M2 groups or between MHC II M2 and MHC II non-M2 groups was determined by Student’s *t* test: *** p < 0.001.

Having observed the natural anti-SIV antibodies in Mauritian cynomolgus macaues, including capture bred and colony bred animals, we asked whether environment or host factors influence the level of these antibodies. While these antibodies were detected in both the capture bred and colony bred monkeys and the antibody variability showed similar patterns, the capture bred monkeys had higher levels of antibodies to PCS1, PCS7, PCS11 and SIVgag (Fig 4A). This suggests that environmental factors may influence the magnitude of natural antibodies to some SIV antigens. In addition, we observed host-specific patterns when the antibody levels to all the SIV peptides were examined in individual monkeys (Table A in S1 File). Among important host factors underlying immune responses and susceptibility or resistance to HIV/SIV infection, the major histocompatibility complex (MHC) plays an important role in the initiation and regulation of immune responses based on their ability to bind and present viral epitopes [7, 15, 19, 29]. We analyzed the MHC haplotypes of the 108 monkeys (Table A in S1 File) and correlated MHC haplotypes with levels of natural antibodies to each of the SIV peptides (Fig 4B and Table B in S1 File). This was based on a relatively small number of monkeys available for some of the MHC haplotypes (Table B in S1 File). MHC haplotype M2 at MHC I or II was significantly correlated with higher natural antibody responses to PCS2 (Fig 4B and 4C, Table B in S1 File). In addition, MHC II of M1 haplotype showed trends of lower antibody responses to PCS1 (p = 0.0883), PCS2 (p = 0.0833), PCS3 (p = 0.0934) and SIVgag (p = 0.0829) (Table B in S1 File). Moreover, Monkeys with M2 haplotype at MHC I and MHC II trended to have higher antibody responses to PCS10 (p = 0.0772) and PCS12 (p = 0.0691), respectively (Table B in S1 File). These data suggest that host MHC haplotypes may differentially affect natural antibody responses to SIV antigens. We expect that larger sample sizes may provide more insight into the correlations between MHC haplotype and antibody responses.

### Cross induction of non-PCS antibodies by PCS vaccination

**Fig 5:**
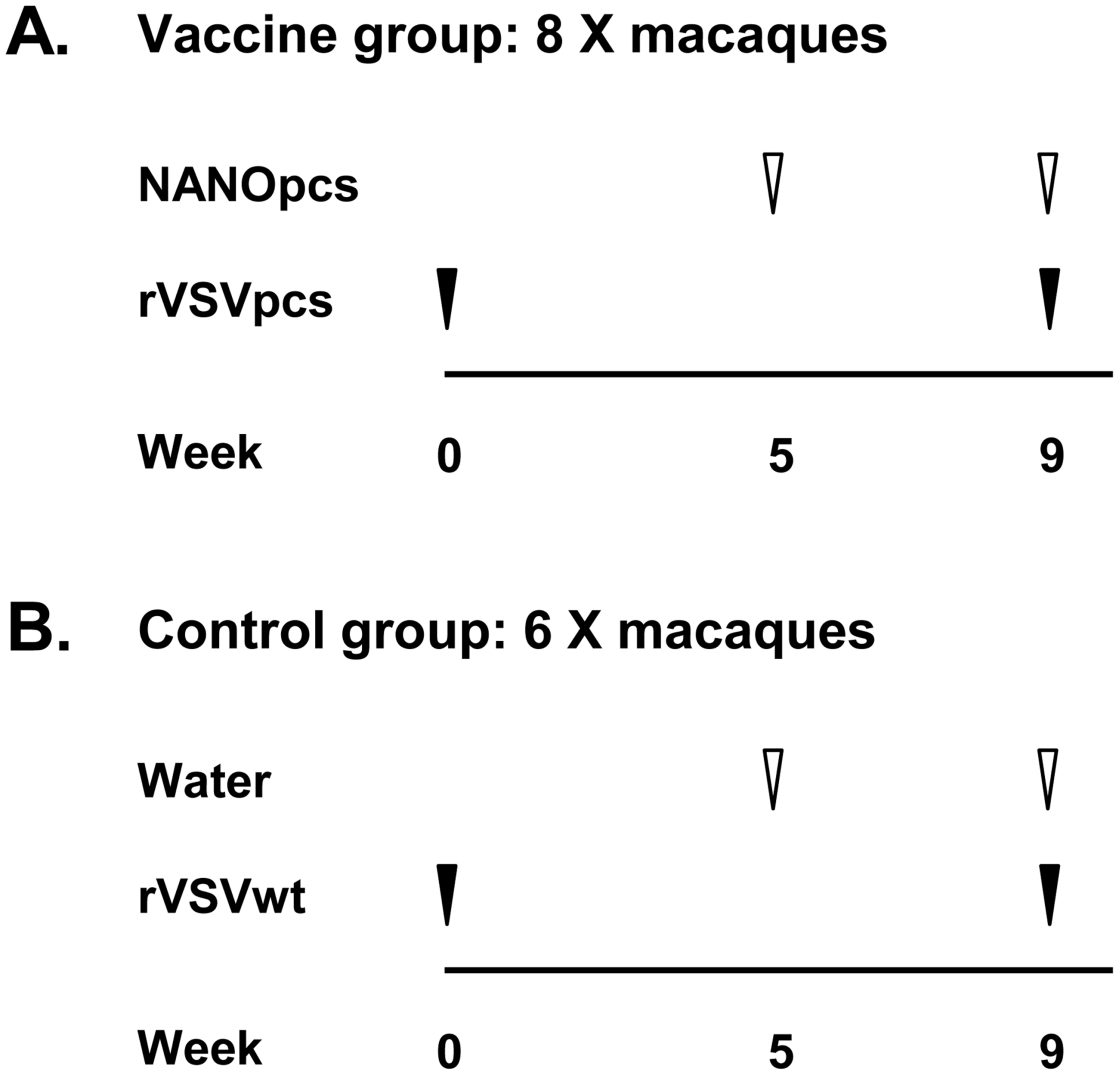
PCS vaccination design. Animals were primed and boosted on indicated dates. rVSVpcs: recombinant vesicular stomatitis viruses (rVSV) expressing peptides derived from SIV protease cleavage sites (PCS peptides), NANOpcs: PCS peptides packaged in nanoparticles (NANO), rVSVwt: rVSV wild type (control virus), and Water: NANO vehicle control. Vaccine production, dose and route of administration are detailed in Materials and Methods.

**Fig 6:**
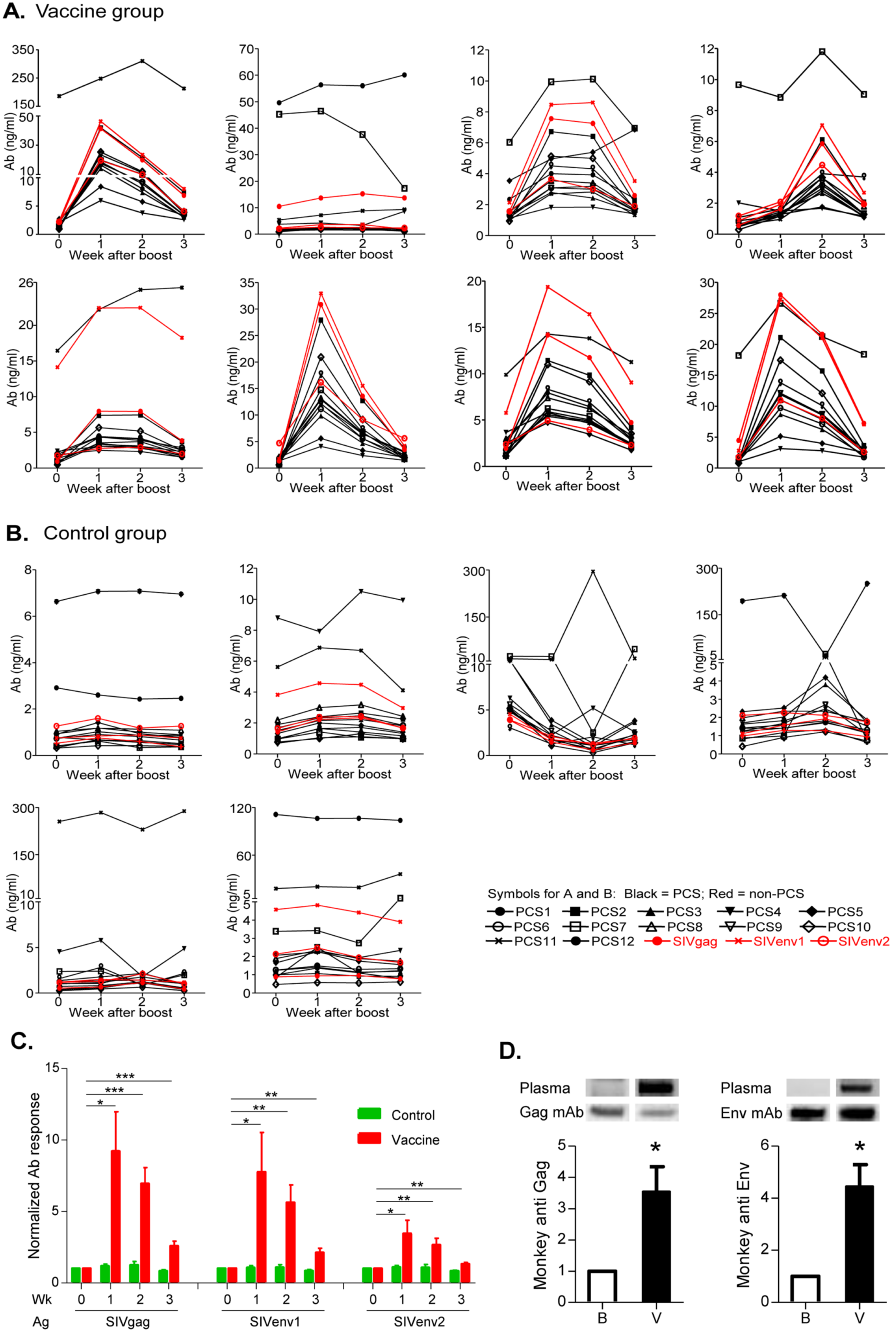
Cross induction of non-PCS antibodies by PCS vaccination. (A) Vaccine group: Eight female Mauritian cynomolgus macaques received a prime with rVSVpcs, first boost with NANOpcs and second boost with rVSVpcs/NANOpcs. Antibodies to SIV peptides were quantified by Bio-Plex. Each panel represents one monkey. Graphs show concentrations of plasma IgG to PCS peptides (black) as well as non-PCS peptides (red) at weeks after the second boost. (B) Control group: Six monkeys received vaccine vehicles including a prime with rVSVwt, first boost with water and second boost with rVSVwt/water, and were analyzed for antibodies. (C) Statistical analysis of antibody responses to non-PCS peptides in A and B. Bars represent normalized mean ± SEM. Student’s *t* test confirmed significant induction of non-PCS antibodies in the vaccine group (* p < 0.05, ** p < 0.01 and *** p < 0.001), but not in the control group. (D) PCS vaccination led to enrichment of monkey plasma antibodies that recognize purified recombinant Gag and Env proteins. Western blot membranes containing recombinant SIV proteins were first probed with plasmas of a PCS-vaccinated monkey. Plasmas from time points of Baseline sampling (indicated as “B” in the graphs) and one week after the second Vaccine boost (indicated as “V” in the graph) were compared for levels of IgG antibodies to the SIV proteins. The membranes were later stripped and re-probed with standard mouse anti-Gag or Env monoclonal antibodies. For quantitative analysis, monkey anti-SIV antibody levels were defined as band intensities with monkey plasma normalized to the corresponding band intensities with standard mouse antibodies. Bar graphs represent mean ± SEM of three independent experiments. Significant induction of antibodies to authentic SIV proteins Gag and Env by PCS vaccination was confirmed by Student’s paired *t* test (* p < 0.05).

In a pilot experiment to evaluate the immunogenicity of the PCS peptides as a novel vaccine candidate (Figs 5 and 6), we observed that, besides their natural occurrence, anti-SIV antibodies could also be nonspecifically induced by vaccination with the vaccine targeting PCS (Fig 6). In the vaccine group (Figs 5A and 6A), eight Mauritian cynomolgus macaques were immunized with PCS peptides delivered by recombinant vesicular stomatitis virus (rVSVpcs) and nanoparticles (NANOpcs). In the control group (Figs 5B and 6B), six animals were immunized with the vaccine vector, recombinant vesicular stomatitis virus wild type (rVSVwt) and water. Antibody responses to SIV peptides were quantified by Bio-Plex antibody assay. As expected, the PCS vaccination elicited antibody responses to the corresponding PCS peptide antigens (Fig 6A, Table 2). However, surprisingly, the PCS vaccination also induced antibodies to all three non-PCS Gag and Env peptides (Fig 6A and 6C, Table 2), although these non-PCS peptides have no sequence overlap with the PCS peptides (Figure A in S1 File). Levels of the non-PCS antibodies largely correlated with those of PCS antibodies (Table 3). Non-specific effect of the vaccine vector (rVSVwt and water) was ruled out since the control group monkeys failed to show the SIV antibody responses (Fig 6B and 6C), while antibodies to VSV (quantified by ELISA) were significantly induced in these control monkeys (Figure B in S1 File). The Bio-Plex SIV antibody data were supported by Western blot showing that PCS vaccination led to the significant increase in monkey antibodies that recognize SIV Gag and Env proteins (Fig 6D). Note that the Env protein does not contain any PCS sequence (Table 1), and the increase in antibodies to Env protein detected by Western blot (Fig 6D) was consistent with the increases in antibodies to non-PCS Env peptides detected by Bio-Plex (Fig 6A–6C). These together confirmed that vaccination against PCS cross-induced antibodies to non-PCS antigens. This observation suggests that a vaccine could lead to “off-target” anti-SIV immune responses that are not directly to the vaccine itself. The potential impact of such immune responses needs to be considered in vaccine design and evaluation as well as result interpretation.

**Table 2:** PCS vaccine cross-induces antibody responses to non-PCS that correlate with antibody responses to PCS.

ID	Wk	PCS1	PCS2	PCS3	PCS4	PCS5	PCS6	PCS7	PCS8	PCS9	PCS10	PCS11	PCS12	SIV gag	SIV env1	SIV env2
AM260	0	1.34	2.07	1.60	2.16	2.74	1.48	1.45	2.11	1.09	0.96	186.17	1.16	2.13	2.31	2.22
	1	16.53	41.94	14.14	5.98	8.43	17.57	19.58	19.88	18.70	25.35	247.65	23.48	41.29	46.33	19.63
	2	8.06	21.59	7.40	3.79	5.76	9.26	10.25	10.04	8.92	12.02	309.93	11.27	20.01	23.44	10.03
	3	3.00	7.45	3.07	2.60	3.36	3.42	3.94	4.12	3.38	3.91	213.00	3.76	6.87	8.02	4.07
AM672	0	49.56	1.36	1.56	3.79	1.67	1.40	45.23	2.02	1.33	0.87	5.40	1.32	10.44	2.27	2.04
	1	56.29	2.20	1.94	4.16	2.49	1.93	46.43	2.48	1.57	2.06	7.16	1.93	13.64	3.57	2.65
	2	55.97	2.33	1.89	3.30	2.14	2.02	37.58	2.60	1.56	1.93	8.82	1.98	15.21	3.62	2.38
	3	60.08	1.16	1.42	8.55	2.12	1.24	17.27	1.76	1.54	1.05	9.32	2.03	13.67	1.88	2.32
AT789	0	2.34	1.32	1.32	1.14	3.54	1.31	6.03	1.53	1.16	0.94	1.00	1.23	1.56	2.13	1.56
	1	4.00	6.71	2.78	1.82	4.96	3.08	9.94	3.55	3.08	5.13	2.69	4.51	7.56	8.46	3.64
	2	3.93	6.42	2.43	1.83	5.39	3.00	10.12	3.40	3.18	5.00	2.82	4.31	7.25	8.60	3.00
	3	2.37	2.14	1.51	1.40	6.85	1.58	6.94	1.67	1.60	1.73	1.32	1.76	2.59	3.53	1.89
AV187	0	0.47	0.52	0.62	2.03	0.56	0.86	9.67	1.08	0.58	0.29	0.60	0.60	0.56	0.87	1.18
	1	1.29	1.33	1.43	1.49	1.29	1.26	8.84	1.64	1.17	1.17	0.96	1.57	1.63	1.93	2.09
	2	2.95	6.11	2.65	1.67	1.76	3.17	11.80	3.64	3.32	3.66	2.82	3.91	5.85	7.04	4.47
	3	1.15	2.07	1.20	1.17	1.09	1.29	9.05	1.80	1.33	1.29	1.14	3.70	2.01	2.68	1.94
AV429	0	0.87	1.23	1.33	2.36	1.85	1.90	0.94	2.20	0.67	0.47	16.44	0.74	0.93	14.12	1.73
	1	3.47	7.38	3.05	3.02	2.47	4.33	3.51	4.42	3.28	5.66	22.20	4.30	7.96	22.42	2.76
	2	2.91	7.44	2.78	3.05	2.32	3.88	3.70	4.15	3.11	5.17	24.97	4.09	7.94	22.46	2.92
	3	1.48	3.72	1.82	2.33	1.54	2.84	1.85	2.52	1.69	2.49	25.27	2.14	3.83	18.26	1.98
BM524	0	2.03	1.32	1.47	1.36	1.98	1.80	0.76	2.11	0.89	0.60	1.97	0.81	1.33	1.69	4.71
	1	12.93	27.91	9.85	4.09	5.60	11.15	14.76	13.15	12.15	20.92	12.05	17.64	30.84	32.95	16.16
	2	6.51	12.75	4.69	2.34	3.32	5.55	6.76	6.60	5.60	9.15	6.44	7.33	13.56	15.53	9.14
	3	2.95	3.76	1.87	1.48	2.03	2.22	1.72	2.47	1.64	1.96	3.18	1.96	3.49	4.20	5.61
BM576	0	2.69	2.05	2.18	3.67	2.52	1.80	1.18	2.97	1.14	1.14	9.88	1.16	1.96	5.78	2.38
	1	7.94	11.42	5.40	5.80	4.71	5.57	6.25	7.41	5.90	11.00	14.28	8.33	14.18	19.35	4.96
	2	6.46	9.84	4.76	4.65	3.48	4.91	5.40	6.20	5.02	9.15	13.81	6.92	11.74	16.42	3.96
	3	2.86	4.20	2.44	2.33	1.76	2.47	2.16	3.34	2.34	3.54	11.25	3.00	4.74	9.06	2.29
BM795	0	1.25	1.27	1.11	1.05	1.98	1.35	18.19	1.53	1.54	0.78	1.57	0.87	4.47	2.80	1.78
	1	11.89	21.09	8.68	3.14	5.14	9.71	26.65	11.04	12.11	17.44	12.07	13.80	28.00	27.26	10.98
	2	8.60	15.71	6.25	2.78	3.98	7.13	21.25	7.86	8.71	12.08	8.55	10.09	21.63	21.01	8.03
	3	2.46	3.65	2.09	1.75	2.52	1.78	18.38	2.54	2.98	2.58	2.60	2.28	7.23	7.04	2.60

Plasma IgG (ng/ml) to SIV peptides at weeks (Wk) after the second boost in PCS-vaccinated monkeys (Fig 6A).

**Table 3:** Pearson correlation analysis of Non-PCS vs PCS antibody levels in Table 2.

Correlation coefficient r value				p value			
	SIVgag	SIVenv1	SIVenv2		SIVgag	SIVenv1	SIVenv2
PCS1	0.3415	-0.1181	0.0027	PCS1	0.0557	0.5196	0.9882
PCS2	0.9117	0.9128	0.9625	PCS2	4.08E-13	3.37E-13	1.53E-18
PCS3	0.9282	0.9071	0.9551	PCS3	2.05E-14	8.41E-13	2.16E-17
PCS4	0.5915	0.4065	0.3547	PCS4	0.0004	0.0210	0.0464
PCS5	0.6766	0.6375	0.6955	PCS5	2.12E-05	8.70E-05	9.92E-06
PCS6	0.9134	0.9299	0.9537	PCS6	3.07E-13	1.43E-14	3.32E-17
PCS7	0.4429	-0.0210	0.1236	PCS7	0.0111	0.9092	0.5003
PCS8	0.9198	0.9166	0.9551	PCS8	1.01E-13	1.77E-13	2.12E-17
PCS9	0.9327	0.8959	0.9495	PCS9	7.95E-15	4.29E-12	1.20E-16
PCS10	0.9208	0.9149	0.9383	PCS10	8.49E-14	2.37E-13	2.27E-15
PCS11	0.3770	0.4033	0.4483	PCS11	0.0334	0.0221	0.0101
PCS12	0.9254	0.9003	0.9530	PCS12	3.57E-14	2.33E-12	4.20E-17

## Discussion

Mauritian cynomolgus macaques are emerging as an attractive NHP species for laboratory research when the traditionally most popular rhesus macaques become limited. Natural immunity to SIV has been documented in a few NHP species [30]. However, this has not been well investigated in cynomolgus macaques. In this study, we screened natural antibody responses to SIV antigens in 108 Mauritian cynomolgus macaques. Our study showed the presence of high levels of natural antibody responses to the SIV antigens including twelve peptides derived from SIV protease cleavage sites (PCS peptides) and three non-PCS Gag or Env peptides, in some of these monkeys. While the impact of these natural antibody responses on susceptibility or resistance to SIV infection remains to be studied, the existence of these anti-SIV antibodies in some of the animals cannot be ignored in HIV vaccine studies using the Mauritian cynomolgus macaque/SIVmac model. Our data provide important information for future vaccine projects. For example, the frequencies of monkeys with substantial antibody responses to SIV antigens can be used to estimate the starting number of monkeys to be screened to select those with low baseline anti-SIV responses. Therefore, such information will facilitate the better use of this important animal model.

Higher antibody responses to some of the SIV peptides were demonstrated in the capture bred group of monkeys than those in the colony bred group, suggesting a possible role of environmental factors such as exposure to environmental stress or other unknown retroviruses. The correlations between monkey MHC haplotypes and antibody responses to specific SIV antigens shown in our study also suggest that host factors may regulate the antibody responses. Since the origin of antigens that induced these antibodies and specific factors and mechanisms that regulate the responses are currently unknown, these topics need to be addressed by extensive future studies.

We observed that monkeys immunized with PCS peptides induced not only antibodies to the PCS peptides but also antibodies to the non-PCS peptides that have no sequence homologies with PCS peptides. There might be two potential explanations based on current knowledge in the field. First, it is possible that although there is no similarity in primary structures between PCS and non-PCS peptides, they may share similar secondary structures that contribute to cross-reactivity [31]. The second possibility could be the presence of endogenous retroviral sequences in primate genomes [32–43]. Some of the endogenous retroviral sequences sharing homology with non-PCS peptides might be expressed in response to stimuli such as vaccination conditions or environmental stress (in the case of natural antibody induction), and result in antibody responses [37, 44–53]. While these possibilities can be explored in future studies, our study showed that a vaccine aimed at specific sites of SIV could hit additional targets. This may potentially impact on the outcome of vaccination and hence need to be considered in vaccine design and evaluation as well as result interpretation.

The term of “off-target effect” was recently used by the vaccine field to describe the phenomena where some vaccines can lead to a variety of unexpected, non-specific responses or outcomes (immunological or non-immunological) other than what are expected based on the intended immune responses specifically targeting the immunogens of interest [54]. The mechanisms appear to be complex and heterogeneous and remain to be understood. Off-target immune responses similar to those observed in our study have not been previously reported in the HIV vaccine field. Part of the reason might be that the classical HIV vaccine approach in most cases used large viral proteins as immunogens and all immune responses targeting the large molecules would be perceived as “on-target”. In our case of testing novel vaccine candidates based on short peptides, we analyzed antibody responses focusing on the short peptides, and included additional non-target peptide antigens in the analysis, making it easier to observe an “off-target” effect.

In conclusion, our findings of natural and vaccine cross-inducible SIV antibodies in Mauritian cynomolgus macaques provide useful new information for HIV vaccine study using this increasingly important NHP model. The presence of these novel immune responses should be considered to achieve better animal selection, experimental design and result interpretation.

## Supporting information

S1 File A single PDF file including Figures A and B and Tables A and B. (PDF)
