# Supplementary material for: Natural and cross-inducible anti-SIV antibodies in Mauritian cynomolgus macaques
Source: PLoS One. 2017 Oct 5;12(10):e0186079. doi: 10.1371/journal.pone.0186079 (PMC5628977; doi:10.1371/journal.pone.0186079)
Supplement: S1 File — (PDF) [file pone.0186079.s001.pdf]

Figure A.

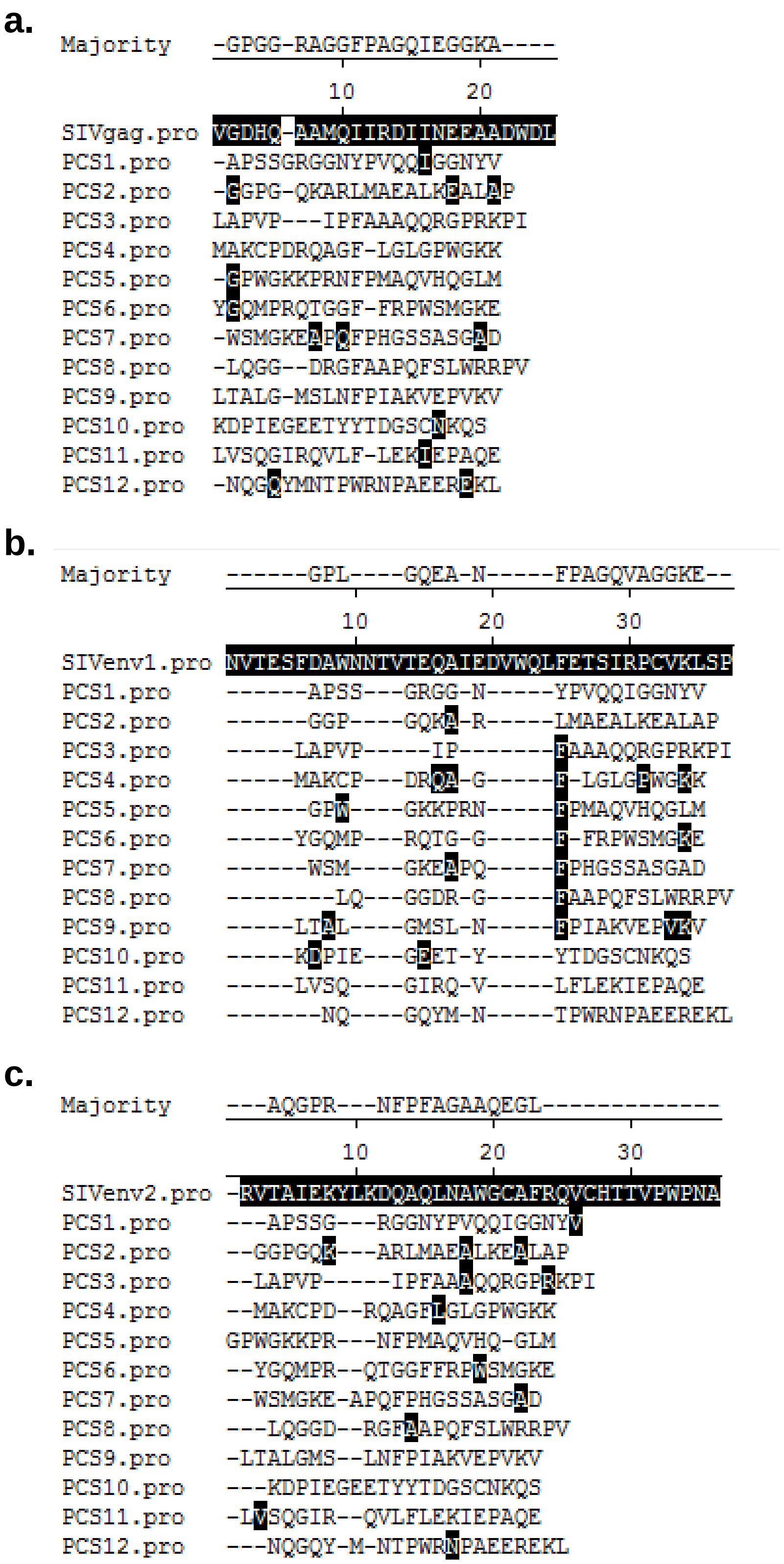

Figure A. Non-PCS peptides have no sequence homology with PCS peptides. The sequences of PCS peptides were aligned against that of non-PCS peptide SIVgag (**a**), SIVenv1 (**b**) or SIVenv2 (**c**) by Clustal W method. Alignment reports are shown, with amino acids that match those of non-PCS peptides decorated in black shades.

Figure B.

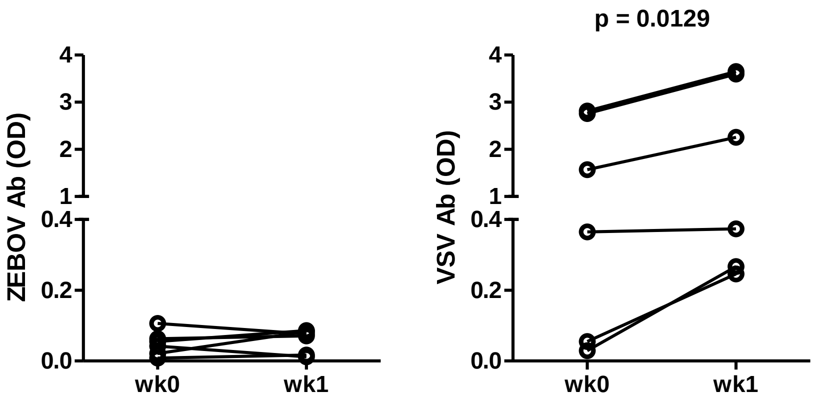

Figure B. rVSVwt induced anti-VSV antibodies in control monkeys of the PCS vaccination experiments. The six control monkeys of the PCS vaccination experiments (i.e. monkeys from Fig 6B), who received vaccine vehicles, rVSVwt and water, were quantified by ELISA for levels of plasma IgG to Zaire Ebola virus (ZEBOV) and vesicular stomatitis virus (VSV) at time points of weeks 0 and 1 after the second boost. OD, optical density. rVSVwt significantly boosted antibodies to VSV but not ZEBOV as confirmed by Student's paired *t* test.

Table A. Information list of 108 female Mauritian Cynomolgus macaques for screening of natural antibodies to SIV antigens.

No.	ID	Source	MHC haplotypes		Antibody (ng/ml)														
			Class I	Class II	PCS1	PCS2	PCS3	PCS4	PCS5	PCS6	PCS7	PCS8	PCS9	PCS10	PCS11	PCS12	SIVgag	SIVenv1	SIVenv2
1	AB484J	Colony bred	M3/M6	M3/M6	3.08	5.15	3.78	4.65	3.14	2.96	4.50	2.95	3.89	3.23	56.10	3.38	3.63	4.34	3.02
2	AB841J	Colony bred	M4/recM4M1	M4/M1	4.24	7.68	4.28	4.54	5.38	6.58	8.53	4.74	5.06	3.14	40.76	6.15	5.69	12.59	5.24
3	AC177I	Colony bred	M1/M3	M1/M3	0.95	1.03	0.79	1.46	1.10	0.89	0.92	1.14	0.93	0.64	0.89	1.17	1.11	1.61	1.33
4	AD241H	Colony bred	M1/M4	M1/M4	0.74	0.62	2.21	1.08	0.77	0.58	0.36	0.76	0.42	0.19	0.44	0.40	0.53	0.55	1.07
5	AE205I	Colony bred	M3/M3	M3/M1	0.41	0.57	0.76	2.88	1.01	0.89	6.62	0.62	0.93	0.49	1.45	0.49	0.40	0.70	1.45
6	AE916H	Colony bred	M1/M3	M1/M3	3.82	4.45	5.56	5.27	3.44	2.64	3.70	3.02	4.10	5.35	3.07	3.78	2.95	5.97	3.23
7	AE969H	Colony bred	M1/M1	M1/M1	3.14	1.33	1.24	5.67	2.28	1.97	1.41	1.94	0.93	0.55	1.93	0.96	3.30	1.42	3.26
8	AF203G	Colony bred	M1/M6	M1/M6	1.57	1.03	1.54	1.02	0.86	0.92	0.93	1.32	0.99	1.20	0.80	1.23	1.14	0.86	1.16
9	AF527H	Colony bred	M1/M4	M1/M4	0.55	0.59	0.58	8.85	13.82	0.65	1.38	0.79	0.36	0.28	0.43	0.56	0.37	1.24	8.10
10	AF855F	Colony bred	recM2M3/M6	M3/M2	1.51	3.05	1.53	1.25	1.42	3.61	1.20	2.06	1.18	0.93	4.59	2.06	2.07	3.51	1.72
11	AH595F	Colony bred	M3/M3	M3/M3	1.16	1.59	1.08	1.31	1.21	2.04	0.89	1.51	1.42	0.90	2.62	1.17	1.38	1.94	1.42
12	AJ753E	Colony bred	M1/M3	M1/M3	1.87	3.28	4.16	4.42	32.84	1.90	2.07	1.59	2.66	3.14	7.91	6.98	1.35	3.08	2.76
13	AL397D	Colony bred	M1/recM4M5	M2/M5	0.55	0.63	0.70	3.25	0.95	0.73	0.50	1.32	0.66	0.43	0.44	0.61	0.65	1.07	13.27
14	AL554E	Colony bred	M5/recM2M1	M5/M2	1.33	3.24	2.24	5.42	2.37	3.30	1.48	2.80	1.49	1.41	3.30	1.85	2.36	3.32	136.33
15	AL66B	Colony bred	M2/recM2M4	M2/M4	3.91	3.30	3.33	1.19	1.63	4.55	2.53	3.16	2.48	3.63	4.92	4.25	4.04	9.61	1.94
16	AL793C	Colony bred	M1/M1	M1/M1	1.27	2.13	1.23	2.10	1.01	1.39	0.74	2.28	0.96	0.81	1.13	1.38	1.38	2.83	1.14
17	AL850A	Colony bred	M1/M6	M1/M4	0.68	1.21	0.87	3.46	1.24	0.95	1.23	1.17	0.68	0.52	0.74	0.84	1.44	3.39	2.95
18	AN190C	Colony bred	M1/M4	M1/M4	1.60	0.54	0.84	1.61	0.96	1.01	0.59	0.84	0.50	0.43	1.70	0.70	0.64	0.93	1.66
19	AP101B	Colony bred	M3/M6	M3/recM6M4M7	1.23	1.03	1.01	5.74	1.16	1.85	0.83	1.11	0.90	0.79	105.01	0.93	0.79	1.23	1.24
20	AP159A	Colony bred	M7/M1	M7/M3	2.58	0.73	0.84	1.11	2.19	1.05	0.50	0.96	0.63	0.67	66.25	14.12	0.70	0.84	1.07
21	AP215A	Colony bred	M3/M6	M2/recM6M4M7	0.59	0.79	1.11	1.12	0.95	0.98	0.67	0.92	0.69	0.73	0.89	0.79	1.07	1.48	1.01
22	AP489B	Colony bred	M3/recM4M1	M3/M1	1.84	0.47	0.40	0.60	0.53	0.41	0.33	0.55	0.25	0.31	0.33	0.31	0.36	0.55	0.50
23	AR939A	Colony bred	M4/M7	M4/M7	2.62	0.44	0.61	1.25	0.98	0.53	1.07	0.73	0.41	0.25	0.41	0.49	0.34	0.58	0.96
24	BF440D	Colony bred	M6/recM3M2	M6/M2	0.96	1.33	1.36	1.49	1.93	1.10	9.51	1.14	1.05	0.79	1.13	9.82	0.76	1.88	1.60
25	BF282E	Colony bred	M3/M4	M3/M4	2.10	1.16	1.53	1.16	1.72	0.89	0.95	1.26	1.25	1.02	1.19	1.08	1.85	4.81	1.66
26	BF289D	Colony bred	M4/recM3M1	M4/M1	0.64	0.44	0.40	1.73	1.01	0.55	0.44	0.55	0.36	0.25	0.36	0.43	0.49	0.73	14.50
27	BF420E	Colony bred	M1/M3	M1/M3	1.21	3.08	1.97	3.83	2.22	1.84	1.13	1.73	2.01	0.93	1.93	1.50	1.21	1.79	2.93
28	BF472D	Colony bred	M3/recM4M2	M3/M2	18.98	0.50	0.55	1.11	1.04	0.68	0.41	0.70	0.45	0.36	1.05	0.55	0.40	0.93	1.24
29	BF513E	Colony bred	M2/M4	M2/M4	0.59	0.76	0.93	1.25	1.10	0.68	0.58	1.08	0.66	0.67	80.71	0.68	1.50	1.02	1.13
30	BF519F	Colony bred	M2/recM3M2	M2/M2	1.30	0.65	0.70	1.36	1.19	1.36	0.53	0.90	0.47	0.39	1.24	0.67	0.95	0.84	7.94
31	BF601C	Colony bred	M1/M6	M1/M6	0.84	0.41	0.46	1.05	0.68	0.52	0.36	0.67	0.28	0.22	0.39	0.40	0.34	4.76	0.67
32	BF615C	Colony bred	M1/M4	M1/M4	1.96	2.91	1.79	1.99	1.39	6.22	3.47	2.30	2.10	2.00	16.04	2.43	3.01	3.99	1.90
33	BF651B	Colony bred	M2/recM1M3	M2/M3	2.93	2.48	3.72	1.65	1.76	1.90	2.33	2.67	2.56	2.77	1.90	2.77	2.56	3.33	1.69
34	BF656B	Colony bred	M6/M4	M6/M1	1.45	0.73	0.90	3.24	1.69	1.21	0.53	1.85	0.57	0.44	238.55	0.76	0.73	0.90	12.52
35	BF664C	Colony bred	M3/M4	M3/M4	1.35	1.55	2.40	1.93	21.13	1.19	1.29	1.38	1.42	1.97	0.83	1.20	0.84	3.19	2.64
36	BF690D	Colony bred	M1/M2	M1/M2	2.50	0.84	0.64	1.96	0.98	0.77	1.16	0.80	0.57	0.50	0.73	0.80	0.74	1.79	5.30
37	BF757C	Colony bred	M3/M6	M2/M6	1.45	1.65	2.09	2.66	7.60	26.34	1.72	1.64	1.34	1.61	0.98	1.56	1.20	2.15	7.27
38	BF919C	Colony bred	M3/M3	M3/M3	0.93	0.81	0.96	2.91	1.13	0.74	0.70	0.92	0.97	0.64	0.71	0.81	0.76	1.24	2.31
39	BF996A	Colony bred	recM1M2/recM4M5	M2/M5	6.49	8.87	10.83	12.45	5.81	3.73	6.90	5.08	6.94	13.13	4.59	7.05	5.17	7.57	3.93
40	BF9F	Colony bred	M2/M3	M2/M3	0.95	0.82	0.83	0.84	0.71	1.30	0.53	0.93	0.79	0.76	1.39	0.96	0.81	1.07	1.14
41	BM52B	Colony bred	M1/recM4M3	M1/M3	1.69	1.46	1.79	2.94	1.66	1.93	1.16	2.15	1.28	0.96	1.72	52.03	1.82	1.87	2.13
42	BM654B	Colony bred	M1/M3	M1/M1	0.92	2.17	0.96	3.21	2.03	1.07	0.92	1.17	0.93	0.79	0.86	0.90	1.05	2.89	2.49
43	JR2E	Colony bred	M2/M5	M2/M5	0.59	5.34	0.49	2.10	0.74	0.80	2.00	0.73	0.45	0.28	1.56	0.64	2.95	0.67	1.66
44	M62J	Colony bred	M1/recM3M2	M1/M2	2.07	0.53	0.64	0.99	1.04	0.79	0.86	0.74	0.60	0.64	13.69	0.64	0.58	1.05	1.48
45	N917K	Colony bred	M4/M7	M4/M7	1.33	1.39	1.88	2.23	2.99	1.87	1.79	1.53	1.76	1.36	2.28	1.39	1.20	1.69	2.64
46	AM466B	Colony bred	M2/M3	M2/M3	2.04	2.84	2.16	1.88	2.28	1.41	2.64	1.96	2.56	1.26	1.72	2.30	1.85	4.62	3.05
47	BF843A	Colony bred	M1/M1	M1/M1	0.56	0.76	0.64	1.77	0.89	5.60	0.59	0.84	0.94	0.52	7.70	0.81	0.84	1.64	3.48
48	270GI	Colony bred	recM3M1/recM4M1	M1/M1	0.89	1.46	1.69	2.38	1.99	1.24	1.67	1.51	2.14	1.13	0.87	1.02	0.90	1.51	4.95
49	AA587K	Colony bred	M4/recM5M1	M4/M4	0.47	0.71	0.65	2.36	0.92	1.04	10.00	0.96	0.45	0.30	0.61	0.61	0.53	4.83	1.53
50	AE938G	Colony bred	M2/M5	M2/M5	0.62	5.43	1.04	1.83	1.27	1.21	0.76	1.30	0.72	0.49	1.79	0.79	0.90	1.69	1.30
51	AF391K	Colony bred	M2/recM2M5	M1/M5	0.33	0.39	0.53	0.91	1.04	6.04	0.31	0.73	0.36	0.24	0.37	0.40	1.60	0.56	0.99
52	AF801F	Colony bred	M1/M1	M1/M1	1.32	2.20	1.72	3.27	1.78	1.48	1.50	1.48	1.52	1.17	4.52	1.47	1.36	2.19	1.61
53	AH121H	Colony bred	M3/M3	M3/M3	2.47	0.69	0.77	1.03	0.74	0.62	0.52	0.80	0.66	0.41	0.74	0.61	0.53	0.74	0.87

54	AK101C	Colony bred	M3/M6	M3/M6	0.89	1.68	0.89	28.73	51.19	1.54	1.20	1.39	0.90	0.41	0.87	1.02	0.86	2.70	2.41
55	AK143F	Colony bred	M1/M5	M1/M5	0.65	0.65	0.71	1.02	0.74	0.92	0.55	0.98	0.60	0.44	0.81	0.56	0.64	2.01	1.05
56	AK178F	Colony bred	M2/M2	M2/M2	3.56	6.38	3.56	4.26	3.53	3.61	5.38	3.36	5.13	2.68	5.51	5.10	5.13	5.04	3.33
57	AK492H	Colony bred	M1/recM4M1	M1/M1	0.30	0.32	0.39	0.79	0.59	0.65	0.28	0.59	0.31	0.22	0.43	0.31	0.33	0.39	1.44
58	AK751E	Colony bred	recM2M6/M2	M6/M2	0.79	0.48	0.62	1.09	0.98	0.73	0.37	0.81	0.51	0.53	0.73	0.55	0.56	0.74	1.50
59	AK820D	Colony bred	recM2M1/M2	M1/M3	0.67	0.50	0.74	1.46	0.87	1.19	0.34	2.04	0.57	0.30	0.40	0.59	0.33	0.62	8.77
60	AK944F	Colony bred	M2/recM2M1	M2/M1	0.33	6.33	0.74	2.16	0.80	1.07	1.32	0.93	15.77	0.30	1.08	0.58	0.46	0.89	1.23
61	AL871C	Colony bred	M2/M4	M2/M4	5.07	7.39	9.87	7.86	7.20	4.74	5.72	4.47	5.62	9.41	4.83	6.40	3.16	5.59	4.40
62	AL891C	Colony bred	M1/M5	M1/M5	0.95	0.69	0.86	1.49	0.77	0.83	3.87	0.98	0.72	0.61	2.28	0.79	0.80	2.31	1.05
63	AM414E	Colony bred	M1/M2	M1/M1	0.36	0.57	0.46	1.00	0.62	0.53	0.46	0.65	0.36	0.27	0.52	0.43	0.40	0.50	0.81
64	AM541D	Colony bred	M2/M3	M2/M3	1.42	2.20	3.01	2.54	1.72	1.30	1.70	1.51	1.67	2.55	1.02	1.64	1.16	3.64	1.76
65	AN984C	Colony bred	recM1M4/M1	M4/M1	1.30	1.48	3.04	7.65	2.73	3.54	1.42	4.50	1.42	1.01	113.94	1.70	1.21	2.58	71.76
66	AT555A	Colony bred	M1/M2	M1/M2	0.50	0.54	0.70	4.79	0.80	1.13	0.58	0.89	1.21	0.39	0.65	2.74	0.47	0.74	1.17
67	BF117F	Colony bred	M4/M4	M4/M4	0.65	2.71	0.68	1.15	0.92	0.77	0.81	0.93	0.65	0.39	112.31	0.73	0.56	1.10	1.17
68	GA138H	Colony bred	M1/recM2M1	M1/M3	0.76	0.71	0.46	1.03	0.62	0.70	0.44	0.65	0.48	0.33	0.81	1.41	0.47	0.56	0.64
69	BF325D	Colony bred	M2/M2	M2/M2	1.33	16.38	0.83	2.08	1.16	2.13	1.35	1.23	0.66	0.53	2.59	0.81	0.73	1.54	3.66
70	BF71G	Colony bred	M1/M5	M1/M5	2.40	4.05	2.95	46.41	2.18	3.56	3.08	2.36	25.05	2.13	3.81	15.39	3.24	3.70	24.00
71	BF790B	Colony bred	M2/M3	M2/M3	0.47	0.53	0.56	1.53	0.56	0.79	0.33	0.92	0.45	0.36	0.61	0.52	0.62	1.17	2.09
72	BF989C	Colony bred	M1/M3	M1/M3	0.65	0.73	0.56	0.76	0.73	1.51	0.68	1.02	0.45	0.39	1.20	0.58	0.56	1.04	2.44
73	BM38B	Colony bred	M1/M3	M1/M3	0.64	0.99	0.68	4.56	2.83	2.06	0.52	1.10	0.63	0.27	0.86	0.64	0.77	1.48	183.14
74	BM48B	Colony bred	M2/M3	M2/M3	11.61	18.01	18.43	13.27	11.70	6.95	14.93	8.96	15.79	17.60	9.88	13.24	9.61	14.61	9.38
75	E143NB	Colony bred	M1/M2	M1/M2	5.04	1.40	1.39	1.77	1.45	4.00	0.95	1.81	1.08	0.83	6.21	1.14	1.39	1.75	7.94
76	F823FH	Colony bred	M1/M4	M1/M4	0.30	0.39	0.40	0.68	0.56	0.49	0.50	0.56	0.56	0.30	0.67	0.40	0.37	0.83	0.90
77	GA828A	Colony bred	M1/M3	M1/M3	0.44	0.47	0.56	1.16	14.25	0.86	0.47	0.61	0.48	0.36	0.55	0.55	0.39	0.59	1.57
78	GA916A	Colony bred	recM4M6/M4	recM6M4/M4	0.33	0.48	0.50	26.54	1.39	0.56	0.33	0.74	0.31	0.27	0.36	0.37	0.83	0.62	1.23
79	GR35BI	Colony bred	M1/M4	M1/M4	0.92	1.08	1.42	3.82	2.73	1.78	1.32	2.01	0.84	0.68	0.84	0.99	0.96	1.30	3.19
80	GR503K	Colony bred	M1/M3	M1/M3	0.84	0.66	0.80	1.49	1.02	1.01	0.84	1.01	0.63	0.39	0.90	0.61	0.59	1.16	2.18
81	GR924L	Colony bred	M3/recM1M4	M3/M4	0.83	1.00	0.90	3.79	1.13	24.40	0.74	1.16	0.85	0.55	1.67	2.12	0.73	5.24	1.11
82	H701AK	Colony bred	M5/recM2M3M1	M5/M1	1.42	1.55	2.30	5.03	2.31	1.44	1.23	2.00	5.25	2.01	97.27	1.35	0.84	1.29	2.01
83	J438CL	Colony bred	M1/M4	M1/M4	0.77	0.82	0.98	1.77	0.98	0.95	0.93	0.98	0.76	0.56	1.29	1.32	0.62	0.92	1.26
84	JR19C	Colony bred	M1/M4	M1/M4	3.08	0.47	0.61	1.48	1.20	1.81	44.86	0.77	0.45	0.33	0.79	0.52	0.50	1.01	2.68
85	LP84B	Colony bred	M3/M1	M3/M2	0.71	0.71	0.77	3.67	1.42	0.73	0.46	0.98	0.57	0.41	0.58	0.67	0.89	2.22	1.29
86	BF541C	Colony bred	M1/M6	M1/M6	4.89	0.47	0.56	0.97	0.77	0.44	0.37	0.61	0.42	0.30	1.13	0.44	1.26	0.65	1.24
87	RAM1GAD	Colony bred	recM4M6/M4	recM6M4/M4	2.34	3.59	2.52	2.69	2.96	2.28	2.71	3.26	2.36	1.72	3.30	3.01	2.53	2.15	2.37
88	S23K	Colony bred	M1/M4	M1/M4	0.86	0.75	0.83	2.04	1.39	0.86	0.90	1.02	0.84	0.39	1.23	0.73	0.64	0.89	1.14
89	S309CG	Colony bred	M4/M4	M4/M4	3.59	0.39	0.39	6.48	0.46	0.50	0.34	0.59	0.36	0.22	0.43	0.31	0.30	0.62	8.46
90	SZ48B	Colony bred	M4/recM1M2	M4/M2	0.41	0.51	0.74	1.27	0.68	0.44	0.34	0.56	0.48	0.46	12.47	0.46	0.21	0.56	23.10
91	T392AG	Colony bred	M3/recM3M4	M1/M4	0.33	0.75	0.59	2.32	0.67	0.62	0.37	0.77	0.41	0.30	89.57	0.49	0.52	0.56	0.84
92	V67J	Colony bred	M1/M1	M1/M1	1.90	3.19	3.81	6.22	2.68	1.82	6.56	2.00	2.45	2.52	1.91	1.96	1.50	2.68	2.96
93	YR335N	Colony bred	M1/recM4M6	M1/recM6M4M7	0.33	0.42	0.50	60.96	1.04	0.67	0.28	0.62	0.87	0.27	0.46	0.49	0.36	0.74	1.05
94	Z518DD	Colony bred	recM1M5/M3	M5/M1	1.42	0.59	0.62	0.97	1.04	0.81	0.49	0.77	0.45	0.33	0.55	0.46	0.49	1.01	1.32
95	AM260	Capture bred	M1/M4	M1/M4	1.05	1.41	1.36	2.13	3.39	1.44	1.60	1.90	0.74	0.59	186.46	1.25	2.45	1.77	1.84
96	AM672	Capture bred	M1/M6	M1/M6	36.68	1.84	1.86	2.18	2.68	2.36	33.77	2.42	1.58	1.21	8.01	2.04	13.58	2.64	2.41
97	AT789	Capture bred	M1/M3	M1/M3	2.06	0.79	0.98	1.12	4.28	0.91	7.62	1.17	0.62	0.53	0.60	0.85	1.27	1.56	1.72
98	AV187	Capture bred	M7/M4	recM7M3/M4	0.49	0.45	0.70	2.12	0.66	0.68	6.80	0.75	0.40	0.33	0.52	0.94	0.63	0.59	0.85
99	AV429	Capture bred	M1/M2	M1/M2	0.96	1.03	1.46	2.72	2.08	2.50	2.42	2.16	0.68	0.43	13.52	0.92	1.20	15.65	1.82
100	BM524	Capture bred	M2/M6	M2/M6	3.25	0.74	1.24	0.96	1.70	1.20	0.82	1.60	0.64	0.46	1.69	0.68	1.17	0.98	4.92
101	BM576	Capture bred	M1/M3	M1/M5	2.12	1.32	1.59	3.12	6.09	1.51	1.09	2.22	0.80	0.70	10.12	1.02	1.45	3.39	1.60
102	BM795	Capture bred	M3/M4	M3/M4	0.68	0.73	0.80	0.57	1.75	1.24	3.98	0.89	0.70	0.48	1.35	0.59	2.16	1.72	1.28
103	AM271	Capture bred	M3/M5	M3/M4	5.43	0.54	0.98	0.75	1.00	0.74	0.47	1.22	0.56	0.36	7.45	0.51	0.58	0.57	1.64
104	AT45	Capture bred	M1/recM1M3	M1/M3	1.60	1.25	1.36	7.61	1.61	1.19	0.87	1.91	0.69	0.70	5.11	0.84	1.27	7.68	1.71
105	BM796	Capture bred	M3/M4	M3/M4	1.47	2.13	5.29	2.60	6.42	2.07	80.35	2.28	2.40	1.76	10.27	1.35	10.20	2.03	2.25
106	BM805	Capture bred	M1/M4	M2/M4	1.04	1.20	1.62	1.33	2.72	1.00	0.93	1.91	0.98	0.48	148.85	0.65	1.05	0.89	1.98
107	BM843	Capture bred	M3/M4	M3/M4	0.48	0.61	1.10	5.83	1.45	0.96	2.45	1.16	0.40	0.36	264.26	0.87	0.60	1.21	1.61
108	BM280	Capture bred	M3/M3	M3/M3	70.81	0.83	1.22	1.48	2.10	1.89	5.99	2.05	0.81	0.63	12.70	1.80	1.20	3.97	1.92

Table B panel a. Univariate analysis of the effects of MHC haplotypes on natural antibodies to SIV PCS1.

MHC I haplotype	No.(%) of monkeys		P value[a]
	Ab high	Ab low	
M1	12 (24)	38 (76)	
Non-M1	14 (32)	30 (68)	0.4898
M2	9 (38)	15 (62)	
Non-M2	18 (24)	57 (76)	0.2013
M3	11 (29)	27 (71)	
Non-M3	17 (28)	43 (72)	1.0000
M4	7 (23)	23 (77)	
Non-M4	20 (30)	46 (70)	0.6256
M5	2 (25)	6 (75)	
Non-M5	27 (28)	68 (72)	1.0000
M6	4 (29)	10 (71)	
Non-M6	25 (28)	65 (72)	1.0000
M7	2 (50)	2 (50)	
Non-M7	28 (27)	76 (73)	0.3080

MHC II haplotype	No.(%) of monkeys		P value[a]
	Ab high	Ab low	
M1	12 (21)	46 (79)	
Non-M1	18 (36)	32 (64)	0.0883
M2	12 (36)	21 (64)	
Non-M2	18 (24)	57 (76)	0.2437
M3	12 (31)	27 (69)	
Non-M3	18 (26)	50 (74)	0.6597
M4	9 (26)	26 (74)	
Non-M4	21 (30)	49 (70)	0.8192
M5	3 (25)	9 (75)	
Non-M5	27 (28)	69 (72)	1.0000
M6	4 (36)	7 (64)	
Non-M6	25 (27)	67 (73)	0.4989
M7	2 (67)	1 (33)	
Non-M7	28 (28)	73 (72)	0.1991

a. Fisher's exact test (uncorrected p value).

Recombinant haplotypes for MHC I or MHC II were excluded from analysis.

Ab high, ≥ 2 ng/ml; Ab low, < 2 ng/ml.

Table B panel b. Univariate analysis of the effects of MHC haplotypes on natural antibodies to SIV PCS2.

MHC I haplotype	No.(%) of monkeys		P value[a]
	Ab high	Ab low	
M1	9 (18)	41 (82)	
Non-M1	14 (32)	30 (68)	0.1515
M2	11 (46)	13 (54)	
Non-M2	14 (19)	61 (81)	0.0137
M3	9 (24)	29 (76)	
Non-M3	17 (28)	43 (72)	0.6470
M4	6 (20)	24 (80)	
Non-M4	20 (30)	46 (70)	0.3326
M5	4 (50)	4 (50)	
Non-M5	22 (23)	73 (77)	0.1084
M6	2 (14)	12 (86)	
Non-M6	25 (28)	65 (72)	0.3483
M7	0 (0)	4 (100)	
Non-M7	28 (27)	76 (73)	0.5707

MHC II haplotype	No.(%) of monkeys		P value[a]
	Ab high	Ab low	
M1	11 (19)	47 (81)	
Non-M1	17 (34)	33 (66)	0.0833
M2	14 (42)	19 (58)	
Non-M2	14 (19)	61 (81)	0.0160
M3	10 (26)	29 (74)	
Non-M3	18 (26)	50 (74)	1.0000
M4	7 (20)	28 (80)	
Non-M4	21 (30)	49 (70)	0.3517
M5	5 (42)	7 (58)	
Non-M5	23 (24)	73 (76)	0.2916
M6	1 (9)	10 (91)	
Non-M6	25 (27)	67 (73)	0.2826
M7	0 (0)	3 (100)	
Non-M7	28 (28)	73 (72)	0.5618

a. Fisher's exact test (uncorrected p value).

Recombinant haplotypes for MHC I or MHC II were excluded from analysis.

Ab high, ≥ 2 ng/ml; Ab low, < 2 ng/ml.

Table B panel c. Univariate analysis of the effects of MHC haplotypes on natural antibodies to SIV PCS3.

MHC I haplotype	No.(%) of monkeys		P value[a]	MHC II haplotype	No.(%) of monkeys		P value[a]
	Ab high	Ab low			Ab high	Ab low	
M1	6 (12)	44 (86)		M1	8 (14)	50 (86)	
Non-M1	11 (25)	33 (75)	0.1159	Non-M1	14 (28)	36 (72)	0.0934
M2	7 (29)	17 (71)		M2	10 (30)	23 (70)	
Non-M2	12 (16)	63 (84)	0.2313	Non-M2	12 (16)	63 (84)	0.1195
M3	9 (24)	29 (76)		M3	9 (23)	30 (77)	
Non-M3	11 (18)	49 (82)	0.6094	Non-M3	13 (19)	55 (81)	0.6281
M4	6 (20)	24 (80)		M4	8 (23)	27 (77)	
Non-M4	13 (20)	53 (80)	1.0000	Non-M4	14 (20)	56 (80)	0.8010
M5	3 (38)	5 (62)		M5	4 (33)	8 (67)	
Non-M5	19 (20)	76 (80)	0.3629	Non-M5	18 (19)	78 (81)	0.2597
M6	2 (14)	12 (86)		M6	2 (18)	9 (82)	
Non-M6	19 (21)	71 (79)	0.7300	Non-M6	19 (21)	73 (79)	1.0000
M7	0 (0)	4 (100)		M7	0 (0)	3 (100)	
Non-M7	22 (21)	82 (79)	0.5799	Non-M7	22 (22)	79 (78)	1.0000

a. Fisher's exact test (uncorrected p value).

Recombinant haplotypes for MHC I or MHC II were excluded from analysis.

Ab high, ≥ 2 ng/ml; Ab low, < 2 ng/ml.

Table B panel d. Univariate analysis of the effects of MHC haplotypes on natural antibodies to SIV PCS4.

MHC I haplotype	No.(%) of monkeys		P value[a]	MHC II haplotype	No.(%) of monkeys		P value[a]
	Ab high	Ab low			Ab high	Ab low	
M1	25 (50)	25 (50)		M1	30 (52)	28 (48)	
Non-M1	20 (45)	24 (55)	0.6842	Non-M1	24 (48)	26 (52)	0.8471
M2	9 (38)	15 (62)		M2	14 (42)	19 (58)	
Non-M2	42 (56)	33 (44)	0.1592	Non-M2	40 (53)	35 (47)	0.4036
M3	19 (50)	19 (50)		M3	17 (44)	22 (56)	
Non-M3	31 (52)	29 (48)	1.0000	Non-M3	36 (53)	32 (47)	0.4231
M4	15 (50)	15 (50)		M4	18 (51)	17 (49)	
Non-M4	31 (47)	35 (53)	0.8280	Non-M4	34 (49)	36 (51)	0.8377
M5	4 (50)	4 (50)		M5	7 (58)	5 (42)	
Non-M5	47 (49)	48 (51)	1.0000	Non-M5	47 (49)	49 (51)	0.7608
M6	7 (50)	7 (50)		M6	5 (45)	6 (55)	
Non-M6	44 (49)	46 (51)	1.0000	Non-M6	45 (49)	47 (51)	1.0000
M7	2(50)	2 (50)		M7	1 (33)	2 (67)	
Non-M7	52 (50)	52 (50)	1.0000	Non-M7	50 (50)	51 (50)	1.0000

a. Fisher's exact test (uncorrected p value).

Recombinant haplotypes for MHC I or MHC II were excluded from analysis.

Ab high, ≥ 2 ng/ml; Ab low, < 2 ng/ml.

Table B panel e. Univariate analysis of the effects of MHC haplotypes on natural antibodies to SIV PCS5.

MHC I haplotype	No.(%) of monkeys		P value[a]	MHC II haplotype	No.(%) of monkeys		P value[a]
	Ab high	Ab low			Ab high	Ab low	
M1	19 (38)	31 (62)		M1	16 (28)	42 (72)	
Non-M1	12 (27)	32 (73)	0.2829	Non-M1	16 (32)	34 (68)	0.6754
M2	5 (21)	19 (79)		M2	9 (27)	24 (73)	
Non-M2	27 (36)	48 (64)	0.2138	Non-M2	25 (33)	50 (67)	0.6543
M3	16 (42)	22 (58)		M3	14 (36)	25 (64)	
Non-M3	18 (30)	42 (70)	0.2773	Non-M3	21 (31)	47 (69)	0.6702
M4	10 (33)	20 (67)		M4	11 (31)	24 (69)	
Non-M4	23 (35)	43 (65)	1.0000	Non-M4	24 (34)	46 (66)	0.8289
M5	3 (38)	5 (62)		M5	5 (42)	7 (58)	
Non-M5	31 (33)	64 (67)	1.0000	Non-M5	30 (31)	66 (69)	0.5198
M6	4 (29)	10 (71)		M6	4 (36)	7 (64)	
Non-M6	30 (33)	60 (67)	1.0000	Non-M6	30 (33)	62 (67)	1.0000
M7	2(50)	2 (50)		M7	2 (67)	1 (33)	
Non-M7	33 (32)	71 (68)	0.5938	Non-M7	33 (33)	68 (67)	0.2614

a. Fisher's exact test (uncorrected p value).

Recombinant haplotypes for MHC I or MHC II were excluded from analysis.

Ab high, ≥ 2 ng/ml; Ab low, < 2 ng/ml.

Table B panel f. Univariate analysis of the effects of MHC haplotypes on natural antibodies to SIV PCS6.

MHC I haplotype	No.(%) of monkeys		P value[a]	MHC II haplotype	No.(%) of monkeys		P value[a]
	Ab high	Ab low			Ab high	Ab low	
M1	9 (18)	41 (82)		M1	11 (19)	47 (81)	
Non-M1	12 (27)	32 (73)	0.3270	Non-M1	14 (28)	36 (72)	0.3606
M2	8 (33)	16 (67)		M2	11 (27)	22 (73)	
Non-M2	14 (19)	61 (81)	0.1611	Non-M2	14 (33)	61 (67)	0.1362
M3	8 (21)	30 (79)		M3	6 (15)	33 (85)	
Non-M3	16 (27)	44 (73)	0.6327	Non-M3	17 (25)	51 (75)	0.3297
M4	5 (17)	25 (83)		M4	8 (23)	27 (77)	
Non-M4	16 (24)	50 (76)	0.5949	Non-M4	17 (24)	53 (76)	1.0000
M5	2 (25)	6 (75)		M5	4 (42)	8 (58)	
Non-M5	21 (22)	74 (78)	1.0000	Non-M5	21 (31)	75 (69)	0.4672
M6	4 (29)	10 (71)		M6	3 (27)	8 (73)	
Non-M6	20 (22)	70 (78)	0.7333	Non-M6	21 (23)	71 (77)	0.7152
M7	0 (0)	4 (100)		M7	0 (0)	3 (100)	
Non-M7	25 (24)	79 (76)	0.5714	Non-M7	25 (25)	76 (75)	1.0000

a. Fisher's exact test (uncorrected p value).

Recombinant haplotypes for MHC I or MHC II were excluded from analysis.

Ab high, ≥ 2 ng/ml; Ab low, < 2 ng/ml.

Table B panel g. Univariate analysis of the effects of MHC haplotypes on natural antibodies to SIV PCS7.

MHC I haplotype	No.(%) of monkeys		P value[a]
	Ab high	Ab low	
M1	10 (20)	40 (80)	
Non-M1	15 (34)	29 (66)	0.1615
M2	8 (33)	16 (67)	
Non-M2	19 (25)	56 (75)	0.4423
M3	11 (29)	27 (71)	
Non-M3	16 (27)	44 (73)	0.8202
M4	10 (33)	20 (67)	
Non-M4	17 (26)	49 (74)	0.4701
M5	3 (38)	5 (62)	
Non-M5	24 (25)	71 (75)	0.4290
M6	3 (21)	11 (79)	
Non-M6	25 (28)	65 (72)	0.7542
M7	1 (25)	3 (75)	
Non-M7	28 (27)	76 (73)	1.0000

MHC II haplotype	No.(%) of monkeys		P value[a]
	Ab high	Ab low	
M1	12 (21)	46 (79)	
Non-M1	14 (34)	33 (66)	0.1331
M2	10 (30)	23 (70)	
Non-M2	19 (25)	56 (75)	0.6406
M3	12 (31)	27 (69)	
Non-M3	16 (24)	52 (76)	0.4944
M4	11 (31)	24 (69)	
Non-M4	18 (26)	52 (74)	0.6441
M5	4 (33)	8 (67)	
Non-M5	25 (26)	71 (74)	0.7303
M6	3 (27)	8 (73)	
Non-M6	25 (27)	67 (73)	1.0000
M7	0 (0)	3 (100)	
Non-M7	28 (28)	73 (72)	0.5618

a. Fisher's exact test (uncorrected p value).

Recombinant haplotypes for MHC I or MHC II were excluded from analysis.

Ab high, ≥ 2 ng/ml; Ab low, < 2 ng/ml.

Table B panel h. Univariate analysis of the effects of MHC haplotypes on natural antibodies to SIV PCS8.

MHC I haplotype	No.(%) of monkeys		P value[a]
	Ab high	Ab low	
M1	11 (22)	39 (78)	
Non-M1	9 (20)	35 (80)	1.0000
M2	8 (33)	16 (67)	
Non-M2	15 (20)	60 (80)	0.2654
M3	6 (16)	32 (84)	
Non-M3	16 (27)	44 (73)	0.3203
M4	6 (20)	24 (80)	
Non-M4	16 (24)	50 (76)	0.7952
M5	3 (38)	5 (62)	
Non-M5	22 (23)	73 (77)	0.3981
M6	3 (21)	11 (79)	
Non-M6	22 (24)	68 (76)	1.0000
M7	0 (0)	4 (100)	
Non-M7	26 (25)	78 (75)	0.5703

MHC II haplotype	No.(%) of monkeys		P value[a]
	Ab high	Ab low	
M1	14 (24)	44 (76)	
Non-M1	12 (24)	38 (76)	1.0000
M2	9 (27)	24 (73)	
Non-M2	17 (22)	58 (78)	0.6305
M3	9 (23)	30 (77)	
Non-M3	17 (25)	51 (75)	1.0000
M4	7 (20)	28 (80)	
Non-M4	18 (26)	52 (74)	0.6299
M5	5 (33)	7 (67)	
Non-M5	21 (26)	75 (74)	0.1558
M6	2 (18)	9 (82)	
Non-M6	23 (25)	69 (75)	1.0000
M7	0 (0)	3 (100)	
Non-M7	26 (26)	75 (74)	0.5712

a. Fisher’s exact test (uncorrected p value).

Recombinant haplotypes for MHC I or MHC II were excluded from analysis.

Ab high, ≥ 2 ng/ml; Ab low, < 2 ng/ml.

Table B panel i. Univariate analysis of the effects of MHC haplotypes on natural antibodies to SIV PCS9.

MHC I haplotype	No.(%) of monkeys		P value[a]	MHC II haplotype	No.(%) of monkeys		P value[a]
	Ab high	Ab low			Ab high	Ab low	
M1	6 (12)	44 (88)		M1	10 (17)	48 (83)	
Non-M1	8 (18)	36 (82)	0.5629	Non-M1	10 (20)	40 (80)	0.8057
M2	7 (29)	17 (71)		M2	8 (24)	25 (76)	
Non-M2	11 (15)	64 (85)	0.1319	Non-M2	12 (16)	63 (84)	0.4199
M3	7 (18)	31 (82)		M3	8 (20)	31 (80)	
Non-M3	10 (17)	50 (83)	1.0000	Non-M3	12 (18)	56 (82)	0.7983
M4	5 (17)	25 (83)		M4	6 (17)	29 (83)	
Non-M4	11 (17)	55 (83)	1.0000	Non-M4	14 (20)	56 (80)	0.7977
M5	2 (25)	6 (75)		M5	3 (25)	9 (75)	
Non-M5	17 (18)	78 (82)	0.6381	Non-M5	17 (18)	79 (82)	0.6925
M6	1 (7)	13 (93)		M6	1 (9)	10 (91)	
Non-M6	18 (20)	72 (80)	0.4571	Non-M6	18 (20)	74 (80)	0.6844
M7	0 (0)	4 (100)		M7	0 (0)	3 (100)	
Non-M7	20 (19)	84 (81)	1.0000	Non-M7	20 (20)	81 (80)	1.0000

a. Fisher's exact test (uncorrected p value).

Recombinant haplotypes for MHC I or MHC II were excluded from analysis.

Ab high, ≥ 2 ng/ml; Ab low, < 2 ng/ml.

Table B panel j. Univariate analysis of the effects of MHC haplotypes on natural antibodies to SIV PCS10.

MHC I haplotype	No.(%) of monkeys		P value[a]	MHC II haplotype	No.(%) of monkeys		P value[a]
	Ab high	Ab low			Ab high	Ab low	
M1	5 (10)	45 (90)		M1	7 (12)	51 (88)	
Non-M1	6 (14)	38 (86)	0.7500	Non-M1	8 (16)	42 (84)	0.5881
M2	6 (25)	18 (75)		M2	7 (21)	26 (79)	
Non-M2	7 (9)	68 (91)	0.0772	Non-M2	8 (11)	67 (89)	0.2248
M3	5 (13)	33 (87)		M3	5 (13)	34 (87)	
Non-M3	8 (13)	52 (87)	1.0000	Non-M3	9 (13)	59 (87)	1.0000
M4	3 (10)	27 (90)		M4	4 (11)	31 (89)	
Non-M4	10 (15)	56 (85)	0.7487	Non-M4	11 (16)	59 (84)	0.7686
M5	2 (25)	6 (75)		M5	3 (25)	9 (75)	
Non-M5	12 (13)	83 (87)	0.2972	Non-M5	12 (13)	84 (87)	0.3679
M6	1 (7)	13 (93)		M6	1 (9)	10 (91)	
Non-M6	13 (14)	77 (86)	0.6865	Non-M6	14 (15)	78 (85)	0.7686
M7	0 (0)	4 (100)		M7	0 (0)	3 (100)	
Non-M7	15 (14)	89 (86)	1.0000	Non-M7	15 (15)	86 (85)	1.0000

a. Fisher's exact test (uncorrected p value).

Recombinant haplotypes for MHC I or MHC II were excluded from analysis.

Ab high, ≥ 2 ng/ml; Ab low, < 2 ng/ml.

Table B panel k. Univariate analysis of the effects of MHC haplotypes on natural antibodies to SIV PCS11.

MHC I haplotype	No.(%) of monkeys		P value[a]
	Ab high	Ab low	
M1	17 (34)	33 (66)	
Non-M1	19 (43)	25 (57)	0.4003
M2	8 (33)	16 (67)	
Non-M2	27 (36)	48 (64)	1.0000
M3	12 (32)	26 (68)	
Non-M3	25 (42)	35 (58)	0.3939
M4	13 (43)	17 (57)	
Non-M4	24 (36)	42 (64)	0.6514
M5	5 (63)	3 (37)	
Non-M5	35 (37)	60 (63)	0.2561
M6	5 (36)	9 (64)	
Non-M6	35 (39)	55 (61)	1.0000
M7	2 (50)	2 (50)	
Non-M7	39 (38)	65 (62)	0.6335

MHC II haplotype	No.(%) of monkeys		P value[a]
	Ab high	Ab low	
M1	19 (33)	39 (67)	
Non-M1	22 (44)	28 (56)	0.2411
M2	14 (42)	19 (58)	
Non-M2	27 (36)	48 (64)	0.5279
M3	13 (33)	26 (67)	
Non-M3	28 (41)	40 (59)	0.5360
M4	16 (46)	19 (54)	
Non-M4	24 (34)	46 (66)	0.2906
M5	6 (50)	6 (50)	
Non-M5	35 (36)	61 (64)	0.3660
M6	3 (27)	8 (73)	
Non-M6	36 (39)	56 (61)	0.5274
M7	2 (67)	1 (33)	
Non-M7	38 (38)	63 (62)	0.5572

a. Fisher's exact test (uncorrected p value).

Recombinant haplotypes for MHC I or MHC II were excluded from analysis.

Ab high, ≥ 2 ng/ml; Ab low, < 2 ng/ml.

Table B panel I. Univariate analysis of the effects of MHC haplotypes on natural antibodies to SIV PCS12.

MHC I haplotype	No.(%) of monkeys		P value[a]	MHC II haplotype	No.(%) of monkeys		P value[a]
	Ab high	Ab low			Ab high	Ab low	
M1	8 (16)	42 (84)		M1	8 (14)	50 (86)	
Non-M1	9 (20)	35 (80)	0.6022	Non-M1	13 (26)	37 (74)	0.1446
M2	7 (29)	17 (71)		M2	10 (30)	23 (70)	
Non-M2	11 (15)	64 (85)	0.1319	Non-M2	11 (15)	64 (85)	0.0691
M3	6 (16)	32 (84)		M3	9 (23)	30 (77)	
Non-M3	11 (18)	49 (82)	0.7919	Non-M3	11 (16)	57 (84)	0.4429
M4	4 (13)	26 (87)		M4	6 (17)	29 (83)	
Non-M4	13 (20)	53 (80)	0.5703	Non-M4	15 (21)	55 (79)	0.7965
M5	1 (13)	7 (87)		M5	2 (17)	10 (83)	
Non-M5	19 (20)	76 (80)	1.0000	Non-M5	19 (20)	77 (80)	1.0000
M6	4 (29)	10 (71)		M6	3 (27)	8 (73)	
Non-M6	16 (18)	74 (82)	0.4637	Non-M6	17 (18)	75 (82)	0.4430
M7	1 (25)	3 (75)		M7	1 (33)	2 (67)	
Non-M7	20 (19)	84 (81)	1.0000	Non-M7	20 (20)	81 (80)	0.4954

a. Fisher's exact test (uncorrected p value).

Recombinant haplotypes for MHC I or MHC II were excluded from analysis.

Ab high, ≥ 2 ng/ml; Ab low, < 2 ng/ml.

Table B panel m. Univariate analysis of the effects of MHC haplotypes on natural antibodies to SIVgag.

MHC I haplotype	No.(%) of monkeys		P value[a]	MHC II haplotype	No.(%) of monkeys		P value[a]
	Ab high	Ab low			Ab high	Ab low	
M1	6 (12)	44 (88)		M1	7 (12)	51 (88)	
Non-M1	10 (23)	34 (77)	0.1828	Non-M1	13 (26)	37 (74)	0.0829
M2	6 (25)	18 (75)		M2	9 (27)	24 (73)	
Non-M2	11 (15)	64 (85)	0.3495	Non-M2	11 (15)	64 (85)	0.1771
M3	5 (13)	33 (87)		M3	7 (18)	32 (82)	
Non-M3	13 (22)	47 (78)	0.4228	Non-M3	13 (19)	55 (81)	1.0000
M4	7 (23)	23 (77)		M4	8 (23)	27 (77)	
Non-M4	11 (17)	55 (83)	0.5732	Non-M4	12 (17)	58 (83)	0.5989
M5	3 (38)	5 (62)		M5	4 (33)	8 (67)	
Non-M5	16 (17)	79 (83)	0.1623	Non-M5	16 (17)	80 (83)	0.2294
M6	3 (21)	11 (79)		M6	2 (18)	9 (82)	
Non-M6	16 (18)	74 (82)	0.7171	Non-M6	17 (18)	75 (82)	1.0000
M7	0 (0)	4 (100)		M7	0 (0)	3 (100)	
Non-M7	20 (19)	84 (81)	1.0000	Non-M7	20 (20)	81 (80)	1.0000

a. Fisher's exact test (uncorrected p value).

Recombinant haplotypes for MHC I or MHC II were excluded from analysis.

Ab high, ≥ 2 ng/ml; Ab low, < 2 ng/ml.

Table B panel n. Univariate analysis of the effects of MHC haplotypes on natural antibodies to SIVenv1.

MHC I haplotype	No.(%) of monkeys		P value[a]	MHC II haplotype	No.(%) of monkeys		P value[a]
	Ab high	Ab low			Ab high	Ab low	
M1	18 (36)	32 (64)		M1	18 (31)	40 (69)	
Non-M1	15 (34)	29 (66)	1.0000	Non-M1	21 (42)	29 (58)	0.3153
M2	8 (33)	16 (67)		M2	13 (39)	20 (61)	
Non-M2	28 (37)	47 (63)	0.3579	Non-M2	26 (35)	49 (65)	0.6679
M3	16 (42)	22 (58)		M3	15 (38)	24 (62)	
Non-M3	20 (33)	40 (67)	0.3982	Non-M3	23 (34)	45 (66)	0.6776
M4	8 (27)	22 (73)		M4	12 (34)	23 (66)	
Non-M4	27 (41)	39 (59)	0.2529	Non-M4	27 (39)	43 (61)	0.8306
M5	4 (50)	4 (50)		M5	6 (50)	6 (50)	
Non-M5	33 (35)	62 (65)	0.4534	Non-M5	33 (34)	63 (66)	0.3449
M6	7 (50)	7 (50)		M6	5 (45)	6 (55)	
Non-M6	31 (34)	59 (66)	0.3711	Non-M6	33 (36)	59 (64)	0.5289
M7	0 (0)	4 (100)		M7	0 (0)	3 (100)	
Non-M7	39 (38)	65 (62)	0.2943	Non-M7	39 (39)	62 (61)	0.2900

a. Fisher's exact test (uncorrected p value).

Recombinant haplotypes for MHC I or MHC II were excluded from analysis.

Ab high, ≥ 2 ng/ml; Ab low, < 2 ng/ml.

Table B panel o. Univariate analysis of the effects of MHC haplotypes on natural antibodies to SIVenv2.

MHC I haplotype	No.(%) of monkeys		P value[a]	MHC II haplotype	No.(%) of monkeys		P value[a]
	Ab high	Ab low			Ab high	Ab low	
M1	20 (40)	30 (60)		M1	25 (43)	33 (57)	
Non-M1	18 (41)	26 (59)	0.5109	Non-M1	21 (42)	29 (58)	1.0000
M2	11 (46)	13 (54)		M2	15 (45)	18 (55)	
Non-M2	32 (43)	43 (57)	0.8164	Non-M2	32 (43)	43 (57)	0.8350
M3	16 (42)	22 (58)		M3	13 (33)	26 (67)	
Non-M3	25 (42)	35 (58)	1.0000	Non-M3	31 (46)	37 (54)	0.2291
M4	12 (40)	18 (60)		M4	14 (40)	21 (60)	
Non-M4	29 (44)	37 (56)	0.8249	Non-M4	33 (47)	37 (53)	0.5369
M5	3 (38)	5 (62)		M5	5 (42)	7 (58)	
Non-M5	42 (44)	53 (56)	1.0000	Non-M5	42 (44)	54 (56)	1.0000
M6	7 (50)	7 (50)		M6	6 (55)	5 (45)	
Non-M6	39 (43)	51 (57)	0.7742	Non-M6	40 (43)	52 (57)	0.5343
M7	1 (25)	3 (75)		M7	1 (33)	2 (67)	
Non-M7	47 (45)	57 (55)	0.6274	Non-M7	46 (46)	55 (54)	1.0000

a. Fisher's exact test (uncorrected p value).

Recombinant haplotypes for MHC I or MHC II were excluded from analysis.

Ab high, ≥ 2 ng/ml; Ab low, < 2 ng/ml.
